# Solar Lignin Reforming with Tunable Selectivity Using a Hybrid Photocatalyst in Aqueous Solution

**DOI:** 10.1021/jacs.5c11981

**Published:** 2025-11-14

**Authors:** Lu Chen, Yongpeng Liu, Sampurna Mitra, Dongseok Kim, Zhipeng Huang, David M. Vahey, Ariffin Bin Mohamad Annuar, Erwin Reisner

**Affiliations:** ‡ Yusuf Hamied Department of Chemistry, 2152 University of Cambridge, Lensfield Road, Cambridge CB2 1EW, U.K.

## Abstract

The valorization of lignin provides an important means to access sustainable base chemicals, but current approaches are hampered by the lack of selective and scalable systems in aqueous solution. Here, we report a ZnIn_2_S_4_ (ZIS)-based photocatalytic system that directs photogenerated electrons to cocatalysts to control product selectivity by using concentrated solar irradiation. By introducing a phosphonated molecular Ni cocatalyst for hydrogen evolution (NiP), the conversion of the lignin model compound 2-phenoxy-1-phenylethanol shifts from phenol and acetophenone on bare ZIS to H_2_ and 2-phenoxy-1-phenylethanone on the ZIS|NiP system (5 sun). The latter achieves a turnover number (TON_H2_) of up to 120 mol_H2_ mol_NiP_^–1^. Integration of a phosphonated Ni bis­(terpyridine) cocatalyst (NitpyP) in a CO_2_-saturated aqueous solution redirects photogenerated electrons toward syngas (H_2_/CO) production (TON_syngas_ = 48), whereas immobilization of formate dehydrogenase (FDH) produces formate (TON_formate_ = 2200). The hybrid photocatalyst could also convert polymeric lignin, albeit with lower activity (e.g., TON_H2_ = 18 for organosolv lignin). Two days of outdoor testing under real sunlight confirms the system’s robustness under fluctuating environmental conditions, with solar heating (to ∼60 °C) enhancing performance and selectivity. Our findings establish photocatalyst tuning as a strategy to control product selectivity in solar lignin reforming, which can be more widely applied in other photocatalytic reactions in the future.

## Graphical abstract

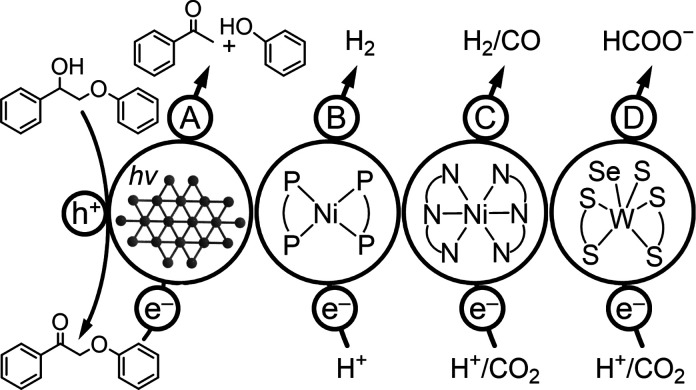

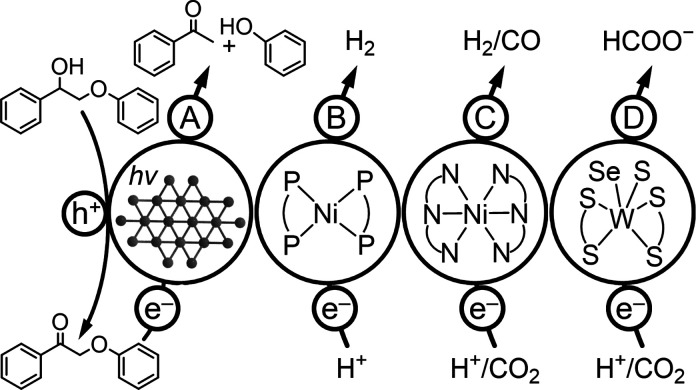

## Introduction

The upcycling of lignocellulosic biomass has emerged as an essential strategy to decarbonize the chemical industry.^1,2^ Lignin, the major renewable aromatic biopolymer, has garnered particular attention for its potential in producing aromatic base chemicals and fuels, but its complex and inert structure make it challenging to valorize.^3^

Solar reforming is a promising approach that can in principle convert lignin into fuel and valuable chemicals under relatively mild conditions.^4–11^ In recent years, several promising photocatalysts, such as CdS,^4,9^ C_3_N_4_,^5,12–14^ and zinc indium sulfide (ZIS),^15–18^ have been developed for the photocatalytic reforming of lignin to H_2_ or value-added chemicals through selective C–O bond cleavage (Table S1). However, control of product selectivity, governed by the fate of photogenerated charge carriers (electrons and holes), is rarely achieved and therefore requires improved strategies. Only a few examples have demonstrated control over product distributions.^19,20^ Many systems also rely on organic solvents for lignin dissolution, which complicates separation, adds cost with enhanced environmental impact and thus hinders scalability.^21–23^

Efficient aqueous phase photocatalysts with high, and ideally controllable, selectivity are therefore needed. (Bio)­molecular electrocatalysts contain metal active sites and offer precise electronic tuning for catalytic activity, which can be exploited in semiconductor-(bio)­molecular catalyst assemblies to achieve selectivity control.^24^

In this study, cocatalysts are employed to direct conduction band electrons in ZIS generated from the photo-oxidation of a lignin model substrate and polymeric lignin, enabling selectivity tuning in aqueous solution. Phosphonic acid groups, as used in NiP ([Ni^II^(P^Ph^_2_{NPhCH_2_P­(O)­(OH)_2_}_2_)_2_]­Br_2_) and phosphonated nickel­(II) bis­(terpyridine) (NitpyP) are particularly effective in promoting aqueous solubility and efficient charge extraction on metal sulfide semiconductors.^25,26^ While photoexcitation of unmodified ZIS predominantly drives C–O cleavage (Pathway A, Scheme 1), combination with a water-soluble molecular H_2_-evolution cocatalyst NiP^27^ directs the photogenerated electrons to proton reduction, enabling highly selective H_2_ production (Pathway B, Scheme 1). Lignin upcycling coupled to CO_2_ utilization can be achieved by combining the ZIS with the CO_2_ reduction cocatalysts NitpyP or the CO_2_ reductase enzyme formate dehydrogenase (FDH), to drive photogenerated electrons to produce syngas or formate (HCOO^–^), respectively (Pathway C or D, Scheme 1).

**Scheme 1:**
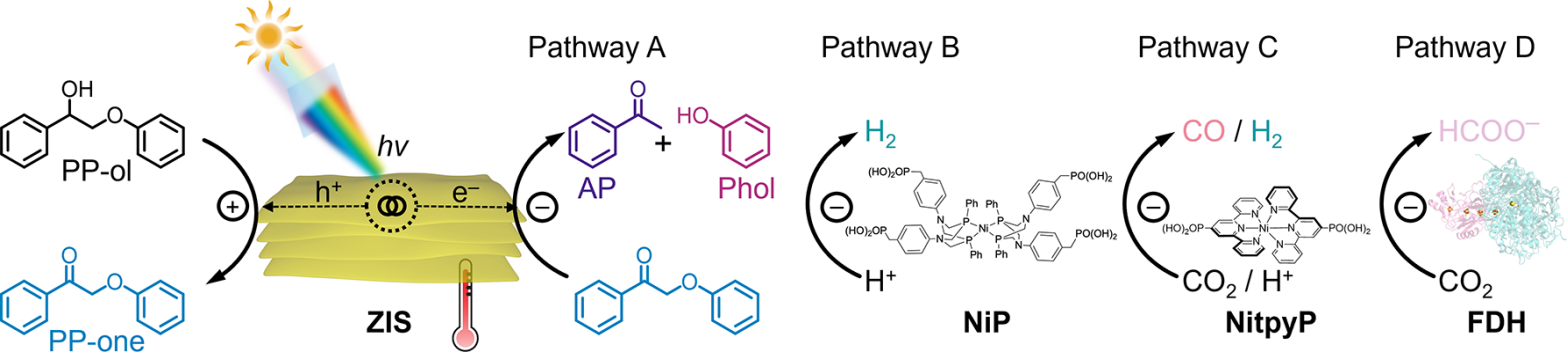
Schematic Illustration of ZIS-Based Photocatalytic Hybrid Systems^a^

a Bare ZIS photocleaves PP-ol to produce AP and Phol through Pathway A. The ZIS|NiP hybrid photooxidizes PP-ol to PP-one with the concomitant reduction of protons to H_2_ through Pathway B. ZIS|NitpyP or ZIS|FDH employs the photogenerated electrons to reduce CO_2_ to CO or HCOO^–^ through Pathway C or D, respectively. Charges for molecular catalysts not shown for clarity.

## Results and Discussion

### Synthesis and Characterization of Photocatalyst

ZIS was synthesized through a low-temperature hydrothermal method at 120 °C (see Supporting Information for experimental details).^15^ Scanning electron microscopy (SEM) and transmission electron microscopy (TEM) images reveal a nanosheet structure of ZIS, and energy-dispersive X-ray (EDX) element mapping confirms the homogeneous distribution of Zn, In, and S (Figure 1a, Figure S1). The X-ray photoelectron spectroscopy (XPS) survey spectrum of ZIS further confirmed the coexistence of Zn, In and S elements (Figure S2). Powder X-ray diffraction (XRD) shows a hexagonal structure in ZIS (Figure S3),^28^ and high-resolution TEM images display lattice fringes with a homogeneous distribution in the electron diffraction ring (Figure 1b), indicating that ZIS is polycrystalline without preferred crystal orientations.

**Figure 1:**
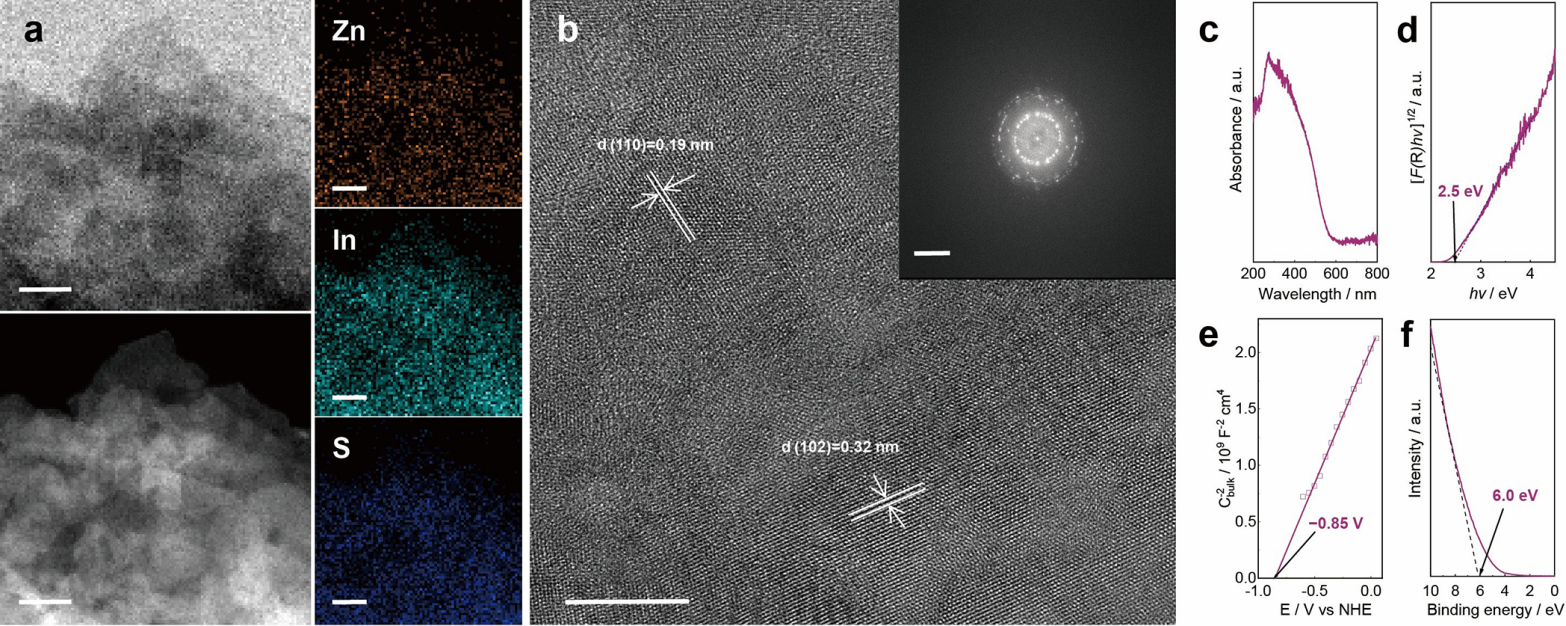
Characterization of the ZIS. (a) Bright field and high-angle annular dark-field image in STEM and EDX mapping (scale bar: 50 nm); (b) high resolution TEM image (scale bar: 10 nm) and the corresponding electron diffraction ring (scale bar: 5 nm^–1^); (c) UV–vis spectrum; (d) Tauc plot; (e) Mott–Schottky plot; (f) UPS spectrum.

The UV–vis spectrum shows a strong absorption in the UV–visible region with an absorption edge at approximately 560 nm (Figure 1c), which corresponds to a band gap of approximately 2.5 eV based on the modified Kubelka–Munk function ([F­(R)­hv]^1/2^ versus photon energy (hv)) (Figure 1d). Mott–Schottky measurements provide insights into the flat-band potential (E_fb_) of ZIS (Figure 1e), which was reported to be approximately 0.15 V more positive^15^ than that of the conduction-band minimum (CBM). Thus, the CBM of ZIS is determined to be −1.0 V vs the normal hydrogen electrode, NHE (E_CB_, pH = 7). The relative location of the valence band maximum (VBM) is 1.56 V vs NHE (E_VB_, pH = 7), according to the 6 eV vs vacuum X-intercept determined by ultraviolet photoelectron spectroscopy (UPS, Figure 1f).

The water-soluble molecular cocatalyst NiP was synthesized as the bromide salt as previously reported (see Supporting Information for synthetic details).^27^ 2-phenoxy-1-phenylethanol (PP-ol), with the most abundant β-O-4 linkages in native lignin,^29–31^ has been selected as the lignin model compound for photocatalytic evaluation.

### Solar Reforming with ZIS and ZIS|NiP

In a typical photoreforming experiment, the photocatalysts (1 mg of ZIS, 100 nmol of NiP) and PP-ol (3 mg) were added in 1 mL of aqueous HCl solution (pH 4; see Figures S4−S5 for optimization), and the mixture was then ultrasonicated to increase the surface area and activity of the photocatalyst. The solar reforming tests were performed under concentrated simulated solar irradiation (5 sun, 500 mW cm^–2^) using a Fresnel lens under a N_2_ atmosphere without any additional sacrificial reagents. Inspired by measurements in gas-phase photothermal catalysis,^32^ the real temperature of the system is measured through an immersed thermometer and infrared thermal imaging (Figure S6). Without external cooling, the reaction temperature stabilized at around 60 °C.

ZIS without cocatalyst photocatalyzed the cleavage of the β-O-4 bond in PP-ol, producing acetophenone (AP) and phenol (Phol) in close to stoichiometric amounts, achieving nearly 90% yields after 6 h (overall quantum yield ϕ_(Phol+AP)_ is 1.2%), with only 5% yield of 2-phenoxy-1-phenylethanone (PP-one) (Figure 2a, Table S2). In contrast, ZIS|NiP achieved a different selectivity, producing H_2_ and PP-one nearly linearly over the first 4 h, reaching 86% and 82% conversions after 6 h (ϕ_(H2)_ is 1.3%) (Figure 2b, Table S3). The stoichiometric amounts of H_2_ and PP-one suggest a photocatalytic mechanism driven by photogenerated electrons and holes (Figure S6, see below). The cooperative interaction between ZIS and NiP enabled this photocatalytic system to achieve an altered selectivity with high activity and a turnover number (TON) of 120 mol_H2_ mol_NiP_^–1^. The cocatalysts themselves are not photoactive, as demonstrated by their negligible visible light absorption in the UV–vis spectra (Figure S7a). Control experiments confirmed that no or only negligible amounts of H_2_ were produced in the absence of ZIS, NiP or light (Table S4). ZIS|NiP shows comparable performance to ZIS|Pt-NPs (Pt photodeposited on ZIS), while it outperformed ZIS modified with other potential cocatalysts such as Ni nanoparticles (ZIS|Ni-NPs), Pd nanoparticles (ZIS|Pd-NPs), ZIS|MoS_2_, ZIS|Ni_2_P, or ZIS in a solution with Ni­(NO_3_)_2_ (Figure 2c, Table S5, Figure S7b,c) (see Supporting Information for experimental details).

**Figure 2:**
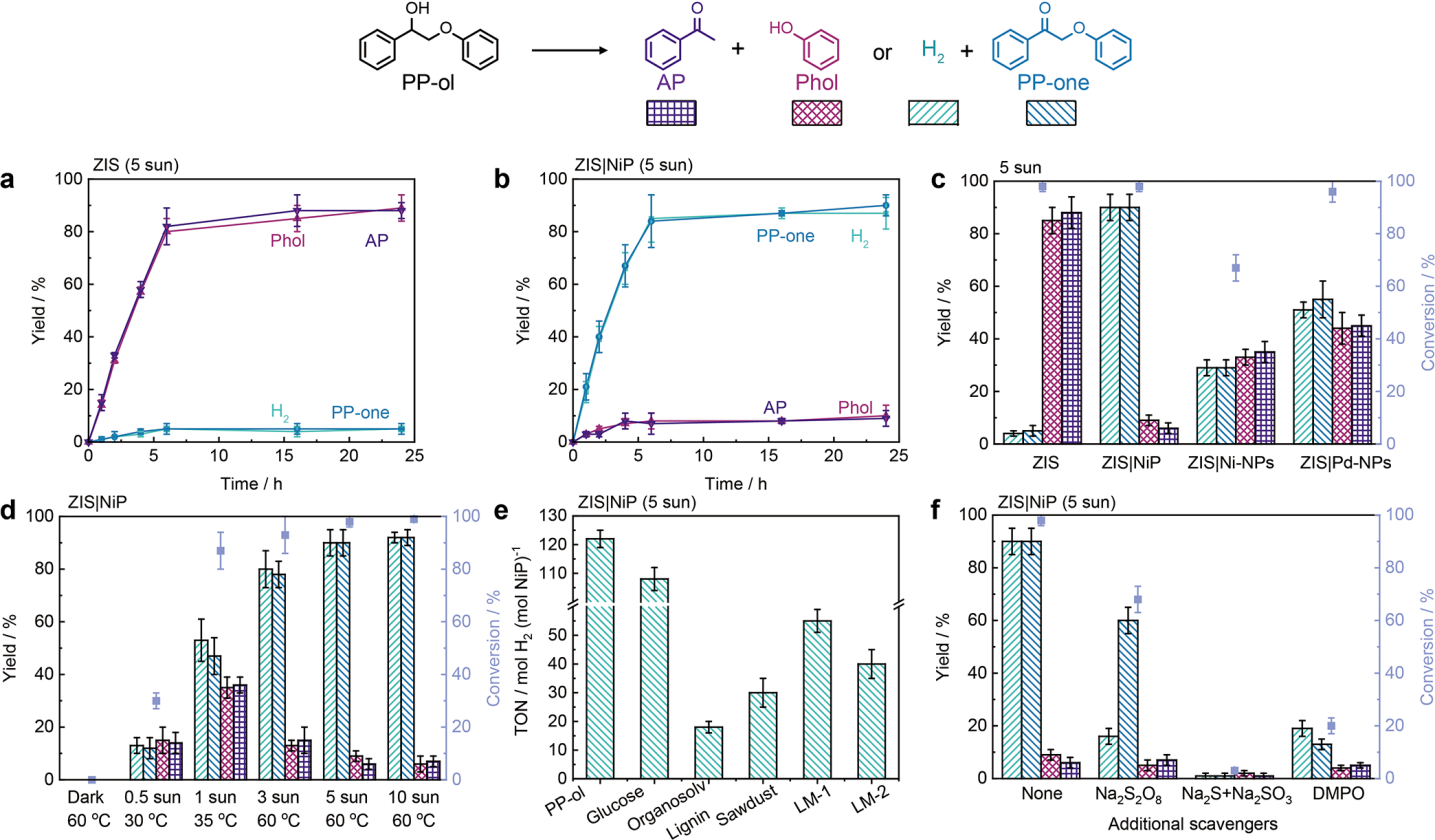
Photocatalytic PP-ol conversion using ZIS and ZIS|NiP. (a) Time profile of PP-ol conversion with ZIS. (b) Time profile of PP-ol conversion with ZIS|NiP. (c) Photocatalytic performance of ZIS, ZIS|NiP and ZIS with other co-catalysts for PP-ol conversion. (d) Effect of light intensity on performance with ZIS|NiP. (e) H_2_ production of ZIS|NiP with different substrates. (f) Activity of ZIS|NiP with different scavengers. Conditions: 1 mg of ZIS, 100 nmol of NiP, 1 mL of HCl aqueous solution (pH 4), 3 mg of PP-ol, 24 h, N_2_ atmophere (with 2% CH_4_), concentrated AM1.5G irradiation (5 sun, 500 mW cm^–2^), 600 rpm stirring. The concentrated sunlight increased the reaction temperature to approximately 60 °C.

Our photocatalysis leverages both the photonic and thermal contributions of sunlight, enabling more efficient solar energy harvesting. Our studies reveal that the activity and selectivity of ZIS|NiP increased with both light intensity and temperature (Figure 2d, Figure S8, Table S6). The reductive cleavage of C–O bonds and reductive production of H_2_ are competing reactions, and the selectivity to H_2_ production is improved under concentrated light. To demonstrate the benefit of solar heat, we studied the activity and selectivity of ZIS|NiP with the solution temperature maintained at 25 °C with external cooling, resulting in significantly lower activity and selectivity (Figure S8).

ZIS|NiP was stable during the reaction, and a slight loss in selectivity was observed with an increased amount of PP-ol: using 15 mg of PP-ol resulted in a TON_H2_ of 505 after 30 h (Figure S9). The stability of the ZIS structure is evidenced by HR-TEM, EDX, XRD, and XPS results (Figure S2–S3), with partially structural change with the formation of In_2_S_3_ after the reaction.^16,17^

The ZIS|NiP system was subsequently studied with various substrates, including glucose, organsolv lignin, sawdust, and two similar lignin model compounds with functional groups (1-(4-hydroxy-3,5-dimethoxyphenyl)-2-(2-methoxyphenoxy)-1,3-propanediol (LM-1) and 1-(3,4-dimethoxyphenyl)-2-(2-methoxyphenoxy)-1,3-propanediol (LM-2)) (Figure 2e, Table S7). The synthesis and characterizations of LM-1, as well as isolation of organsolv lignin from birch chips, can be found in the Supporting Information (Figures S10–S16). The TON_H2_ for glucose reforming is 108, with 5-HMF and gluconic acid as the oxidation products (Figures S17a). Compared with PP-ol, the H_2_ yield of other functionalized lignin model compounds were lower, yielding a TON_H2_ of 55 and 40 for LM-1 and LM-2, respectively. The conversion of real lignin is more challenging due to its complex cross-linked structure, leading to a TON_H2_ of 18 and 30 for organosolv lignin and raw sawdust (oxidation products see Figures S17b), respectively.

To gain insight into the reaction mechanism of the photocatalytic system, we tested the effects of different scavengers. When an electron scavenger (Na_2_S_2_O_8_) was introduced, the conversion of PP-ol decreased from 100 to 70%, with PP-one as the predominant product with 60% yield (Figure 2f, Table S8). The addition of hole scavengers (Na_2_S+Na_2_SO_3_) significantly decreased the conversion of PP-ol to 3%, suggesting that photogenerated holes play a crucial role in the initial oxidation step of PP-ol. These phenomena indicate that both photogenerated electrons and holes participate in lignin photoreforming (Scheme 1). Furthermore, the addition of a radical scavenger, 5,5-dimethyl-1-pyrroline-N-oxide (DMPO), led to a substantial suppression of the reaction, indicating the possible formation of radical intermediates.

Thus, we propose that photoexcitation generates charge carriers in the ZIS semiconductor, with holes facilitating the oxidation of PP-ol to PP-one, followed by the reductive cleavage of the β–O–4 bond by photogenerated electrons in ZIS.^17^ In contrast, photogenerated electrons in ZIS|NiP are directed to the NiP cocatalyst, where they participate in the reduction of protons to form H_2_. The migration of charge carriers is critical in tuning products selectivity. Cleavage of the β–O–4 linkage in PP-ol requires a bond dissociation energy (BDE) of 54–72 kcal mol^–1^.^33^ The oxidation of the α carbon atoms in the β-O-4 linkage can efficiently weaken the C–O bond energy by around 14 kcal mol^–1^, making the cleavage process easier.^34,35^ Consequently, PP-one exhibits a lower BDE than PP-ol, facilitating further C–O bond cleavage. The lower BDE of PP-one was confirmed by using a commercial TiO_2_ photocatalyst (Figure S18), where PP-one showed a conversion rate higher than that of PP-ol. These findings suggest that after solar-driven H_2_ production (with H_2_ separated in the gas phase) the remaining oxidation products can be further processed to produce aromatic compounds via easier C–O bond cleavage.

The applicability of our photocatalytic systems was further validated through outdoor experiments (Figure 3a). The average outdoor temperature and the reaction temperature of day 1 were 18 and 60 °C (from solar heating provided through light concentration by a Fresnel lens), respectively; and of day 2 were 10 and 50 °C, respectively (Figure 3b). The light intensity fluctuated between 0 and 1 sun due to cloud cover, with an averaging light intensity of 0.6 Sun on day 1 and 0.8 Sun on day 2 (Figure S19). Despite variations in temperature and light intensity, the catalytic performance of ZIS|NiP or ZIS remained consistent with the results obtained from standardized indoor experiments (Figure 3c, Table S9). The TON_H2_ with ZIS|NiP remained >100, whereas the selectivity of Phol and AP with ZIS was >90%.

**Figure 3:**
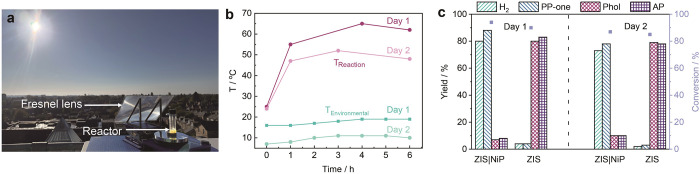
Outdoor experiments on rooftop of Yusuf Hamied Department of Chemistry, University of Cambridge, on 17^th^ September and 11^th^ October 2024. (a) Photographic image of outdoor photocatalytic reactor setup using a Fresnel lens to concentrate sunlight. (b) Environmental temperature and reaction temperature over time. (c) Catalytic performance of ZIS|NiP and ZIS on PP-ol conversion. Conditions: 1 mg of ZIS, 100 nmol of NiP, 1 mL of HCl aqueous solution (pH 4), 3 mg of PP-ol, N_2_ atmosphere (with 2% CH_4_), 6 h solar irradiation, 600 rpm stirring.

### Solar Reforming with CO_2_ Utilization

Next, we conducted studies to tune the selectivity of photoreduction by using different CO_2_ reduction cocatalysts.^36–38^ We aimed to utilize photogenerated electrons for the photocatalytic reduction of a CO_2_-saturated aqueous solution, enabling simultaneous conversion of a solid (lignin) and a gaseous (CO_2_) waste stream into valuable products such as syngas and formate (Figure 4). We tested four molecular metal bis­(terpyridine) based cocatalysts^39,40^ for the photoconversion of PP-ol and CO_2_: a phosphonated nickel­(II) bis­(terpyridine) catalyst (NitpyP) and three cobalt­(II) bis­(terpyridine) complexes with different functional groups at the central 4’-terpyridine ring (R = PO_3_H_2_, NH_2_ and COOH; denoted as CotpyR with R = P, NH_2_ and H). The NitpyP was synthesized by self-assembly of Ni­(BF_4_)_2_·6H_2_O with 2 equiv of phosphonated terpyridine (tpy), 2,2′:6′,2″-terpyridine-4′-phosphonic acid (tpyP).^40^

**Figure 4:**
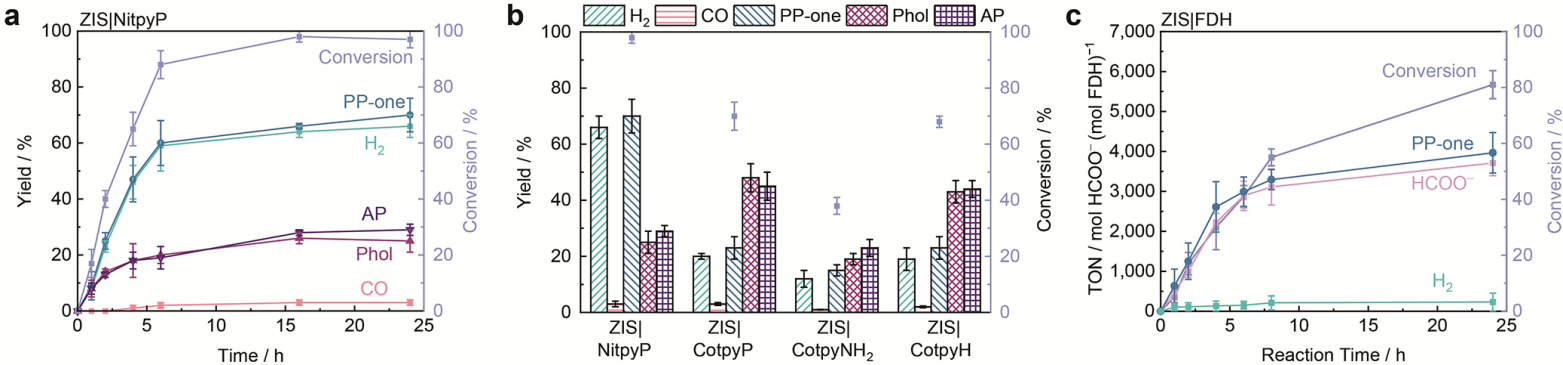
Photocatalytic CO_2_ reduction performance of ZIS|M-tpyR and ZIS|FDH . (a) Time profile of PP-ol conversion with ZIS|NitpyP. (b) Photocatalytic performance of ZIS|MtpyR on PP-ol conversion. Reaction conditions (a,b): PP-ol 3 mg, ZIS (1 mg mL^–1^), MtpyR 200 nmol, CO_2_-saurated 1 mL 0.1 M aqueous NaHCO_3_, 600 rpm stirring and concentrated AM1.5G irradiation (5 sun, 500 mW cm^–2^). (c) Time profile of the ZIS|FDH catalytic system. Reaction conditions: PP-ol 1 mg, ZIS (0.5 mg mL^–1^), FDH 50 pmol, CO_2_-saturated 1 mL 0.1 M aqueous NaHCO_3_ (containing 2% CH_4_), 600 rpm stirring and AM1.5G irradiation (100 mW cm^–2^).

The photogenerated electrons in ZIS were transferred to the MtpyR molecular catalysts, leading to selectivity toward H_2_ and CO (Figure 4b, Table S11). Among the tested catalysts, NitpyP exhibited the highest photocatalytic activity and selectivity, achieving 100% conversion and 70% selectivity toward producing a mixture of H_2_ and CO (TON_syngas_ = 48, ϕ_(H2+CO)_ is 0.9%), a key feedstock for the well-established Fischer–Tropsch process to yield transportation fuels and high-value chemicals. Despite the successful detection of some CO from CO_2_ reduction, H_2_ remained the dominant photoreduction product (Figure 4a, Figure S19, Table S9), as proton reduction is typically more favorable than CO_2_ reduction in aqueous systems.

Furthermore, we explored enzymatic cocatalysis, leveraging the high selectivity, reversibility, and catalytic efficiency of enzymes under moderate overpotentials.^41,42^ [W]-formate dehydrogenase (FDH) from *Nitratidesulfovibrio vulgaris* Hildenborough (*Nv*H) is the model enzymatic electrocatalyst for the conversion of CO_2_, which selectively catalyzes the formation of HCOO^–^, with H_2_O serving as the proton source.^43^ FDH (50 pmol) was used with ZIS (0.5 mg) and PP-ol (1 mg) in a CO_2_-saturated aqueous NaHCO_3_ solution (0.1 M, 1 mL) under an aerobic condition and AM1.5G irradiation (100 mW cm^–2^) at 25 °C to avoid damage of FDH at high temperature.^44^ The ZIS|FDH catalytic system achieved a TON_formate_ of 2200 after 4 h (Figure 4c, Table S12, Figure S21a) (ϕ_(HCOO−)_ is 0.1%). Notably, the amounts of HCOO^–^ and PP-one are closely matched, indicating balanced photogenerated electron and hole consumption (Figure S21b). The activity of FDH started to decrease after 4 h irradiation and was almost completely lost after 8 h. Given the recently reported photostability of FDH for at least 24 h,^41,42,45^ we hypothesize that the pronounced photothermal effects of ZIS, even under conditions in which the bulk temperature of the photocatalytic system is maintained at 25 °C, generate localized heat at the ZIS–FDH interface and may damage the enzyme. Considering the high oxidative potential of photogenerated holes, FDH degradation may also result from direct photooxidative damage by ZIS. These effects are both immediate and sufficiently intense to cause irreversible damage to FDH, thereby compromising the system stability. Isotopic labeling experiments were conducted to unambiguously confirm the carbon source in CO_2_ photoreduction on the ZIS|FDH assembly (Figure S22).

Charge carrier dynamics were investigated using photoelectrochemical impedance spectroscopy (PEIS) with a ZIS photoelectrode (see Experimental Section for details, Figure S23). Upon the incorporation of FDH, R_ct_ decreased from 160.3 ± 11.0 kΩ to 75.6 ± 3.9 kΩ, demonstrating an enhanced charge transfer process facilitated by FDH^46^ as well as the resistive nature of ZIS photoelectrodes in the order of tens of kΩ.^47,48^ Furthermore, the interaction between FDH and ZIS was further investigated using quartz crystal microbalance (QCM) to evaluate the adsorption and desorption processes of FDH on ZIS.^49^ The QCM profile exhibits a rapid adsorption phase lasting approximately 6 min and only 18% of FDH desorbed in the washing step, indicating that the association between FDH and ZIS is both rapid and strong (Figure S24).

### Solar Reforming with Tunable Pathways

To validate the versatility of the solar lignin reforming platform and the tunability of the photocatalytic reactions, we designed a consecutive experiment in which four distinct reaction pathways were selectively activated *in situ* within a photoreactor (Figure 5). The experiment began with pristine ZIS under 5 sun irradiation for 10 h under a N_2_ atmosphere, during which PP-ol was converted into a mixture of AP and Phol products (8.1 ± 0.8 μmol; pathway A). Subsequent introduction of FDH under 1 sun irradiation for 10 h under a CO_2_ atmosphere enabled selective CO_2_ reduction to formate (0.4 ± 0.1 μmol) through pathway D. Then, addition of NitpyP and 5 sun irradiation for 10 h in CO_2_ shifted the CO_2_ reduction products toward syngas formation, yielding 5.1 ± 0.3 μmol H_2_ and 0.2 ± 0.03 μmol CO (pathway C). Finally, acidification of the solution to pH 4 followed by the addition of NiP under 5 sun irradiation for 10 h under N_2_ resulted in H_2_ production (7.3 ± 0.8 μmol; pathway B). Compared with the individual measurements reported previously (Figures 2–4), syngas and H_2_ yields in the one-pot consecutive experiment were approximately 40–60% lower. This decrease is likely attributed to the progressive aging of ZIS, surface blockage of ZIS by denatured FDH, improved mass transfer in the 1 mL individual system compared with the 3 mL one-pot system, the low aqueous solubility of PP-ol, and the nonuniform 5 sun illumination in the larger reaction volume. Collectively, these results highlight the tunable selectivity and versatility of the solar-driven lignin reforming platform for valorizing lignin model compounds through multiple reaction pathways using the same ZIS molecule and solution.

**Figure 5:**
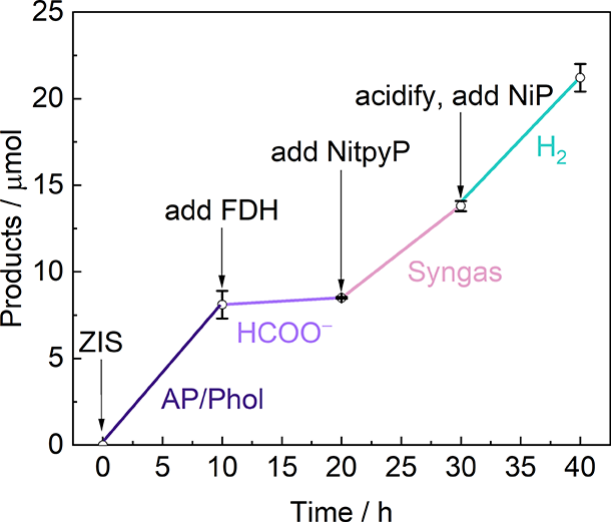
Consecutive solar reforming of a lignin model compound with tunable reaction pathways. Conditions: ZIS (1 mg mL^–1^), FDH (50 pmol mL^–1^), NitpyP (200 pmol mL^–1^), NiP (100 pmol mL^–1^), 3 mL of 0.1 M NaHCO_3_, 600 rpm stirring, N_2_ atmosphere (with 2% CH_4_) for ZIS and ZIS|NiP or CO_2_ atmosphere (with 2% CH_4_) for ZIS|NitpyP and ZIS|FDH, 500 mW cm^–2^ for ZIS, ZIS|NitpyP and ZIS|NiP, and 100 mW cm^–2^ for ZIS|FDH.

## Conclusions

We have established a versatile ZIS-based photocatalytic system that regulates product selectivity in aqueous solution. This ZIS-platform allows steering photogenerated electron pathways through cocatalyst engineering, with activity and selectivity enhanced by solar heating provided by concentrated solar irradiation. While switchable selectivity has previously been realized to give two products using photocatalysis,^50,51^ we establish here the possibility of producing four different products with bespoke cocatalysts from lignin valorization. This strategy opens new possibilities in tailoring fuel and chemical syntheses from complex polymeric substrates using light. Without a cocatalyst, the photogenerated electrons generated on ZIS break C–O bonds selectively. When the photogenerated electrons migrate to a specific (bio)­molecular cocatalyst, it enables photoreduction of protons to H_2_, CO_2_ to syngas or CO_2_ to HCOO^–^ coupled to the formation of a ketone (PP-one), which has a lower BDE than the substrate (PP-ol). Directing electron fate with cocatalysts in photocatalytic processes offers a versatile approach for tuning the products of lignin and CO_2_ conversion, opening possibilities for photocatalysis delivering a versatile range of products on demand. While this work has focused on controlling the reduction pathways, the same conceptual strategy may be applied to modulate the oxidation half-reaction, thereby providing a more complete toolbox for selectivity tuning in the future.

## Supplementary Material



## Data Availability

Raw data that support the findings of this study are available from the University of Cambridge data repository: https://doi.org/10.17863/CAM.122719.

## Notes

Experimental section, material characterization, photocatalysis data, product quantification (nuclear magnetic resonance and mass spectrometry analysis), electrochemical, quartz crystal microbalance and spectroscopy data (Figures S1–S24, Tables S1–S12) (PDF)

## §

Low-Carbon Conversion Science and Engineering Center, Shanghai Advanced Research Institute, Chinese Academy of Sciences, 201210 Shanghai, China
