# Supplementary material for: Solar Lignin Reforming with Tunable Selectivity Using a Hybrid Photocatalyst in Aqueous Solution
Source: J Am Chem Soc. 2025 Nov 14;147(47):43509–16. doi: 10.1021/jacs.5c11981 (PMC12673586; doi:10.1021/jacs.5c11981)
Supplement: Supplementary file 1 [file ja5c11981_si_001.pdf]

Supporting Information

Solar Lignin Reforming with Tunable Selectivity Using a Hybrid Photocatalyst in Aqueous Solution

Lu Chen,[1,2,†] Yongpeng Liu,[1,†] Sampurna Mitra,[1] Dongseok Kim,[1] Zhipeng Huang,[1] David M. Vahey,[1] Ariffin Bin Mohamad Annuar,[1] Erwin Reisner[1,*]

[1]Yusuf Hamied Department of Chemistry, University of Cambridge, Lensfield Road, Cambridge, CB2 1EW, UK
[2]Present Address: Low-Carbon Conversion Science and Engineering Center, Shanghai Advanced Research Institute, Chinese Academy of Sciences, 201210, Shanghai, China

† These authors contributed equally to this work.
* e-mail: reisner@ch.cam.ac.uk

Experimental Section

Materials. $InCl_3\cdot4H_2O$ (99.99%, Thermo Scientific), $Zn(CH_3COO)_2\cdot2H_2O$ (99.5+%, Sigma-Aldrich), thioacetamide (98%, Sigma-Aldrich), $Ni(NO_3)_2\cdot3H_2O$ (98+%, Fisher Scientific), 2-methoxyphenol (99+%, Thermo Scientific), syringaldehyde (98+%, Thermo Scientific), MoS_2 (98%, Thermo Scientific Chemicals) and Ni_2P (98%, Sigma-Aldrich).were used as received without further purification. Gases (N_2 and CO_2) were supplied by BOC.

Physical Characterisation. ^{1}H NMR spectroscopy was recorded on a Bruker DPX 400 MHz spectrometer with the chemical shifts (δ) of the ^{1}H NMR spectra being referenced against the residual solvent signal ($CDCl_3$: δ = 7.26 ppm). Powder XRD patterns were recorded on a Panalytical X'pert Pro diffractometer by using Cu Kα radiation (40 kV, 30 mA) using 1° min^{-1} scan rate. XPS measurements were performed at the Maxwell Centre (University of Cambridge) with a near ambient pressure X-ray photoemission spectroscopy system that uses a SPECS XR 50 MF X-ray Source, μ-FOCUS 600 X-ray monochromator and differentially pumped PHOIBOS 150 1D-DLD near ambient pressure analyser. Data analyses were carried out using CasaXPS software. UV/Vis spectroscopic measurements were performed on a Varian-Cary 5000 spectrophotometer equipped with a diffuse reflectance accessory; spectra were collected with white paper as a reference. A TESCAN MIRA3 field emission gun-scanning electron microscope equipped with an Oxford Instruments Aztec Energy X-maxN 80 EDS system was used for SEM images. TEM and EDX measurements were performed on a FEI Talos F200X G2 S electron microscope operated at an acceleration voltage of 200kV. Samples for TEM measurements were suspended in ethanol and dispersed ultrasonically. Drops of suspensions were dropped on a Holey carbon film on 300 mesh Cu grids.

Synthesis of ZIS. ZIS photocatalysts were synthesized through a low-temperature hydrothermal method. $InCl_3\cdot4H_2O$ (1.0 mmol), $Zn(CH_3COO)_2\cdot2H_2O$ (0.5 mmol), and thioacetamide (2 mmol) were dissolved in water (50 mL). The mixture was heated to 120 °C and maintained at the same temperature for 5 h under vigorous stirring. The solution was then cooled to room temperature naturally, and the precipitate was collected by centrifugation, washed with water and ethanol (abs) three times, and dried under vacuum at 60 °C overnight.

Synthesis of metal complexes. NiP, NitpyP and CotpyR were synthesized and characterized according to previously reported procedures.[1,22,23]

ZIS|M-NPs photocatalysts. ZIS (50 mg), $Ni(NO_3)_2\cdot3H_2O$ (100 nmol), methanol (1 mL) and H_2O (10 mL) were mixed in a 50 mL flask, and the mixed system was purged by N_2 gas for 30 min. The mixture was then illuminated under UV–vis light (300 W Xe lamp) for 2 h under rigorously stirring. The solid was collected by centrifugation and washed with water (3×40 mL), and dried under vacuum at 60 °C overnight.

Synthesis of lignin model compounds. 1-(4-hydroxy-3,5-dimethoxyphenyl)-2-(2-methoxyphenoxy)-1,3-propanediol (LM-1) was synthesized according to reported procedures (see Scheme S1 and synthetic procedures below).

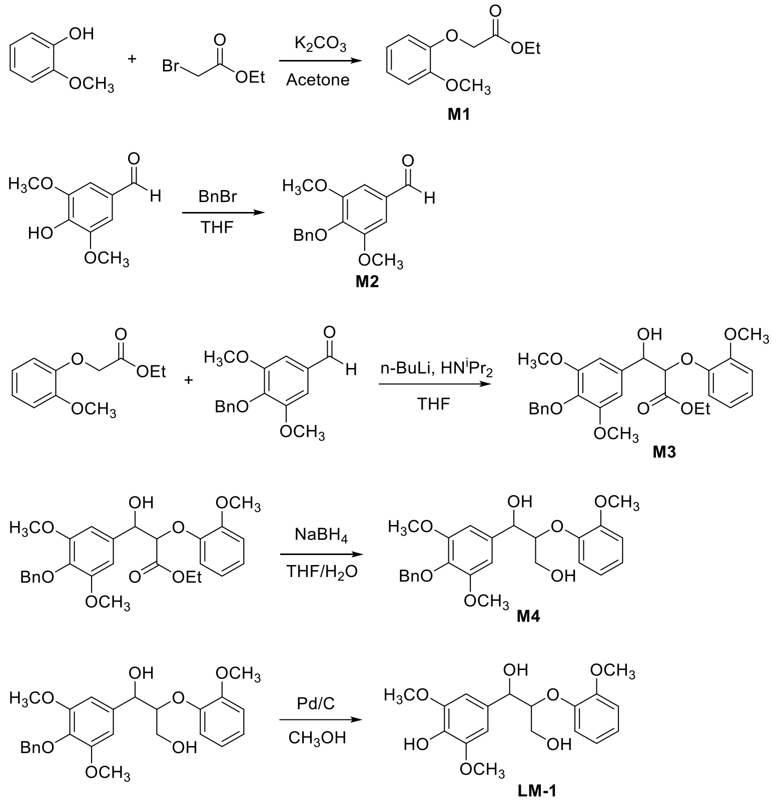

Scheme S1. Scheme for synthesis of 1-(4-hydroxy-3,5-dimethoxyphenyl)-2-(2-methoxyphenoxy)-1,3-propanediol (LM-1).

Ethyl (2-methoxyphenoxy)acetate (M1).[2] Dry K_2CO_3 (1.72 g, 12.5 mmol), 2-methoxyphenol (1.55 g, 12.5 mmol), and acetone (65 mL) were added to a dry, argon-flushed three-necked flask. The mixture was stirred for 15 min and then cooled to 0 °C using an ice bath. After adding ethyl bromoacetate (2.08 g, 12.5 mmol), the reaction mixture was warmed to reflux and stirred overnight. Upon cooling, the solution was filtered through a pad of Celite and washed with acetone. The combined filtrates were concentrated under reduced pressure to near dryness, then diluted with diethyl ether (20 mL). The organic layer was washed sequentially with aqueous NaOH solution (5% w/w, 3 × 25 mL), water (25 mL), and brine (25 mL). After drying over $MgSO_4$, the solution was filtered and concentrated under reduced pressure. The crude residue was purified by flash chromatography (EtOAc/hexanes, 2:1) to afford M1 as a pale yellow oil.

4-(Benzyloxy)-3,5-dimethoxybenzaldehyde (M2).[3] A suspension of syringaldehyde (2.50 g, 13.72 mmol) and potassium carbonate (4.00 g, 14.4 mmol) in THF (25 mL) was treated with benzyl bromide (1.0 mL, 8.2 mmol). The reaction mixture was refluxed for 12 h, cooled to room temperature, and poured into ice-cold water. The aqueous layer was extracted with EtOAc (4 × 25 mL). The combined organic extracts were dried over $MgSO_4$, filtered, and concentrated. Purification of the residue by flash chromatography (EtOAc/hexanes, 3:7) afforded compound M2 as a colorless oil.

Ethyl 3-(4-(benzyloxy)-3,5-dimethoxyphenyl)-3-hydroxy-2-(2-methoxyphenoxy)-propanoate (M3).[4] To a solution of diisopropylamine (0.24 mL, 1.73 mmol) in THF (2.5 mL) was added *n*-BuLi (0.69 mL, 2.5 M solution in hexane, 1.73 mmol) dropwise at 0 °C. The mixture was stirred at this temperature for 20 min and then cooled in a dry ice acetone bath. To the mixture was added a solution of M1 (0.334 g, 1.57 mmol) in THF (2 mL) dropwise, followed by M2 (0.4285 g, 1.57 mmol) in THF (1 mL). The mixture was stirred for 1 h and then quenched by addition of saturated aqueous NH_4Cl

(2 mL). The organic layer was separated and the aqueous layer was extracted with EtOAc (3 x 10 mL). The organic extracts were combined and dried over sodium sulfate, filtered, and the solvent removed under vacuum. To the solid obtained was added diethyl ether (5 mL) and the mixture was stirred until a fine solid formed. The solid was collected by filtration. The mother liquor was concentrated under reduced pressure and purified on silica gel with 3:7 ethyl acetate: hexanes to provide **M3** as a white solid.

1-(4-(benzyloxy)-3,5-dimethoxyphenyl)-2-(2-methoxyphenoxy)propane-1,3-diol (M4).[4] To a solution of M3 (0.553 g, 1.14 mmol) in THF/H_2O (3:1, 4 mL) was added sodium borohydride (0.216 g, 5.71 mmol). After stirring overnight at room temperature, the reaction was quenched with saturated aqueous NH_4Cl (3 mL) and extracted with ethyl acetate (3 × 10 mL). The combined organic extracts were dried over Na_2SO_4, filtered, and concentrated. Purification by flash chromatography (silica gel, 4:6 EtOAc/hexanes) afforded **M4** as a colorless oil.

1-(4-hydroxy-3,5-dimethoxyphenyl)-2-(2-methoxyphenoxy)-1,3-propanediol (LM-1).[2] To a solution of **M4** (0.401 g, 0.90 mmol) in MeOH (4.0 mL) under a nitrogen atmosphere was added Pd/C (30 mg, 10% w/w). The mixture was degassed via three vacuum/H_2 cycles, then stirred under a hydrogen atmosphere (balloon) at room temperature overnight. The catalyst was removed by filtration through Celite, washing thoroughly with MeOH (3 × 5 mL). The combined filtrates were concentrated *in vacuo*, and the residue was purified by flash chromatography (silica gel, 6:4 EtOAc/hexanes) to afford **LM-1** (20% overall yield, over 5 steps).

Photocatalysis experiments. Photocatalytic reactions were conducted in quartz reactors, and a solar light simulator (AM1.5G) with different intensity by a Fresnel lens. ZIS (1 mg), 100 nmol NiP, 1 mL HCl aqueous solution (pH 4), and PP-ol (3 mg) were added to the reactor, and with the aid of sonication, the mixture formed a well-dispersed suspension. Then, the reactor was purged with N_2 gas with CH_4 as an internal standard, and placed under illumination. the reaction mixture was stirred at 600 rpm during the reaction. After the reaction, the generated H_2 was quantified by manual injection from the headspace of the reactors into a Shimadzu GC gas chromatograph. 1 mL acetonitrile was added into an aliquot of the liquid, filtered through a 0.22 mm Nylon syringe filter and analyzed by HPLC. The incident photon number was determined to be $(1.9 \pm 0.2) \times 10^{19}$ photons per hour under simulated AM1.5G irradiation (300−800 nm),[5] and the quantum yield was determined after 6 h irradiation.

The overall quantum yield (ϕ_{overall}) is defined as:

$$\phi\text{overall (\%)} = \frac{2 \times n(products)}{\text{Incident photons}} \times 100$$

Isotopic Labeling. Isotopic labeling experiments were carried out either in a $NaH^{12}CO_3$ (0.1 M) aqueous solution containing PP-ol (3 mg ml^{-1}) with $^{12}CO_2$ as the headspace gas or in a $NaH^{13}CO_3$ (0.1 M) aqueous solution containing PP-ol (3 mg ml^{-1}) with $^{13}CO_2$ as the headspace gas, under simulated AM 1.5G irradiation. ^1H NMR spectra (DMSO-d_6) were collected with a 700 MHz TXO Cryoprobe NMR spectrometer.

ZIS photoelectrode. ZIS was deposited onto an FTO-coated glass substrate through dropcasting. 10 mg ZIS was added in 1 mL isopropanol/H_2O (1:1) and ultrasonicated for 15 min. The glass were cleaned by sonication in isopropanol (2 × 15 min), ethanol (2 x 15 min), and rinsing with H_2O. The FTO side of the glass was covered with a Kapton tape mask leaving 1.0 cm^2 area for deposition. Three

layers of 30 μL of suspension were dropcast on the glass. Then 2 μL Nafion in 30 μL isopropanol were dropcast on the final layer, and it was allowed to dry in air.

Photoelectrochemistry. Linear sweep voltammetry (LSV), cyclic voltammetry (CA), and photoelectrochemical impedance spectroscopy (PEIS) were carried out under AM1.5G irradiation in a photoelectrochemical (PEC) cell with a three-electrode configuration: a ZIS working electrode, a Pt mesh counter electrode, and a Ag/AgCl reference electrode. The working electrode was fabricated by drop-casting ultrasonicated ZIS suspension (5 mg ml^{-1}) on FTO. The CO_2-saturated electrolyte contains $NaHCO_3$ (0.1 M), KCl (50 mM), and PP-ol (3 mg ml^{-1}). The reference electrode potential was converted to reversible hydrogen electrode (RHE) by the Nernst equation. LSV and CA were recorded on an Ivium potentiostat. PEIS was measured on a BioLogic VSP potentiostat with frequency ranges from 1 MHz to 0.5 Hz and a 25-mV sinusoidal amplitude. Impedance data was fitted with equivalent circuits using modeling software ZView2 (Scribner Associates).

Quartz Crystal Microbalance (QCM). QCM experiments were carried out on a Biolin Q-Sense Explorer module and a custom-designed QCM cell within an anaerobic glovebox (MBraun, N_2 atmosphere, <0.1 ppm O_2). A gold-coated quartz chip with a surface area of 0.79 cm^2 and a surface roughness <1 nm RMS was utilized. The chip was initially functionalized by drop casting an ultrasonicated suspension (0.1 ml) of ZIS (0.5 mg ml^{-1}) in isopropanol, forming a thin layer on the surface. To establish a stable baseline, prior to measurements, an enzyme-free aqueous solution (2 ml) containing $NaHCO_3$ (0.1 M) was flowed through the system at a rate of 0.141 ml min^{-1} for a duration of at least 1 h. Once the baseline reached a steady state, 50 pmol of FDH was introduced into the buffer solution (2 mL) for evaluating enzyme loading for 30 min. Subsequently after enzyme loading, a washing process was performed by flowing an enzyme-free aqueous solution (10 ml) containing $NaHCO_3$ (0.1 M) at a rate of 0.141 ml min^{-1} for a duration of 30 min. The adsorption and desorption of the enzyme onto the surface was quantified by monitoring changes in the resonance frequency of the piezoelectric quartz chip. To determine the corresponding mass change, the change in frequency (Δf) was analyzed using the Sauerbrey equation:

$$\Delta f = -\frac{2f_0^2}{A\sqrt{p_q \mu_q}}\Delta m$$

where f_0 is the resonance frequency (5 MHz) of the quartz oscillator, A is the piezoelectrically active crystal area, Δm is the change in mass, p_q is the density of quartz, and μ_q is the shear modulus of quartz. Assuming 25% of the adsorbed mass consisted of water molecules bound to the enzymes, Δm can be converted into quantity of enzymes.

Organosolv birch lignin

For the photocatalytic conversion of organosolv birch lignin, lignin was first extracted from birch sawdust, according to a reported procedure.[6] Briefly, dried birch sawdust (5.0 g) was added to1,4-dioxane (36 mL) containing 2N HCl (4 mL) in a 250 mL flask. The mixture was heated to a gentle reflux under a N_2 atmosphere for 1 hour, and then cooled to room temperature. After filtration, the filtrate was partially concentrated in vacuum to give a gummy residue. The gummy residue was taken up in acetone/water (9:1, ~15 mL) and precipitated by drop-wise addition to rapidly stirring water (1 L). The precipitated solid was collected by filtration and dried under vacuum. The dried crude lignin was taken up in acetone/methanol (9:1) and precipitated by dropwise addition to rapidly stirring Et_2O (1 L). The precipitated lignin was collected by filtration and dried in a freeze dryer for 2 days to give an organosolv birch lignin.

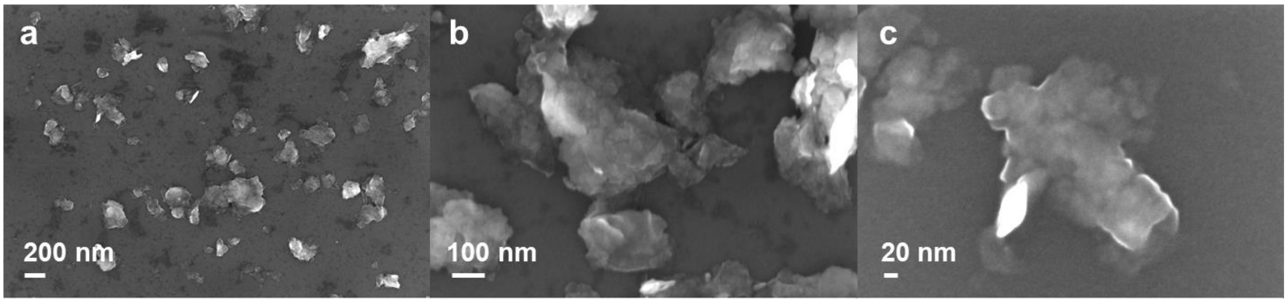

Figure S1. SEM images of ZIS.

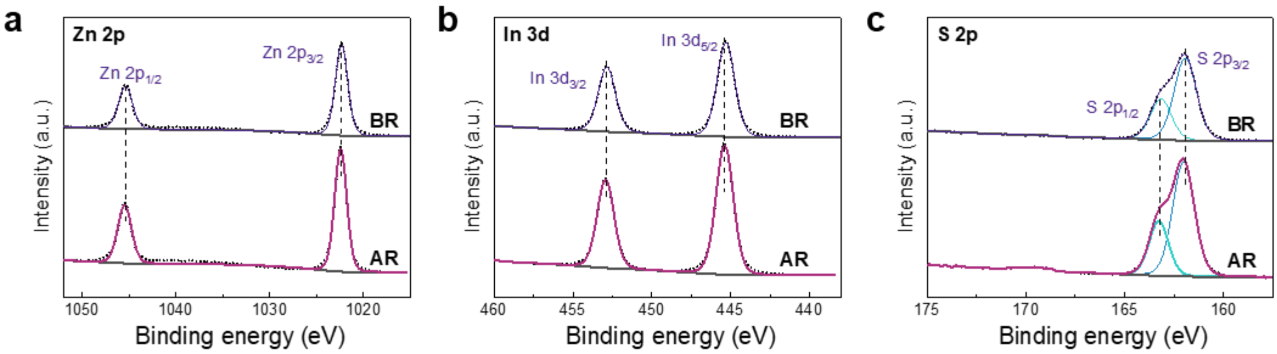

Figure S2. XPS of ZIS (Zn 2p, In 3d and S 2p). The X-ray photoelectron spectroscopy (XPS) survey spectrum of ZIS further confirmed the coexistence of Zn, In and S elements, in agreement with the EDX analysis. (**a**) The Zn 2p spectrum exhibits peaks centred at 1021.34 and 1044.36 eV corresponding to $2p_{3/2}$ and $2p_{1/2}$, respectively, confirming the presence of Zn^{2+}.[7] (**b**) The peaks centred at 444.30 and 452.40 eV are ascribed to In $3d_{5/2}$ and In $3d_{3/2}$, assigning to In^{3+} binding state. (**c**) In the S 2p spectrum, the binding energies of 161.06 and 162.31 eV can be assigned to the S $2p_{3/2}$ and S $2p_{1/2}$, respectively, suggesting the presence of S^{2-}. Notably, the XPS spectra of Zn 2p, In 3d, and S 2p remain unchanged before and after the photoreaction, demonstrating the stability of ZIS under reaction conditions.

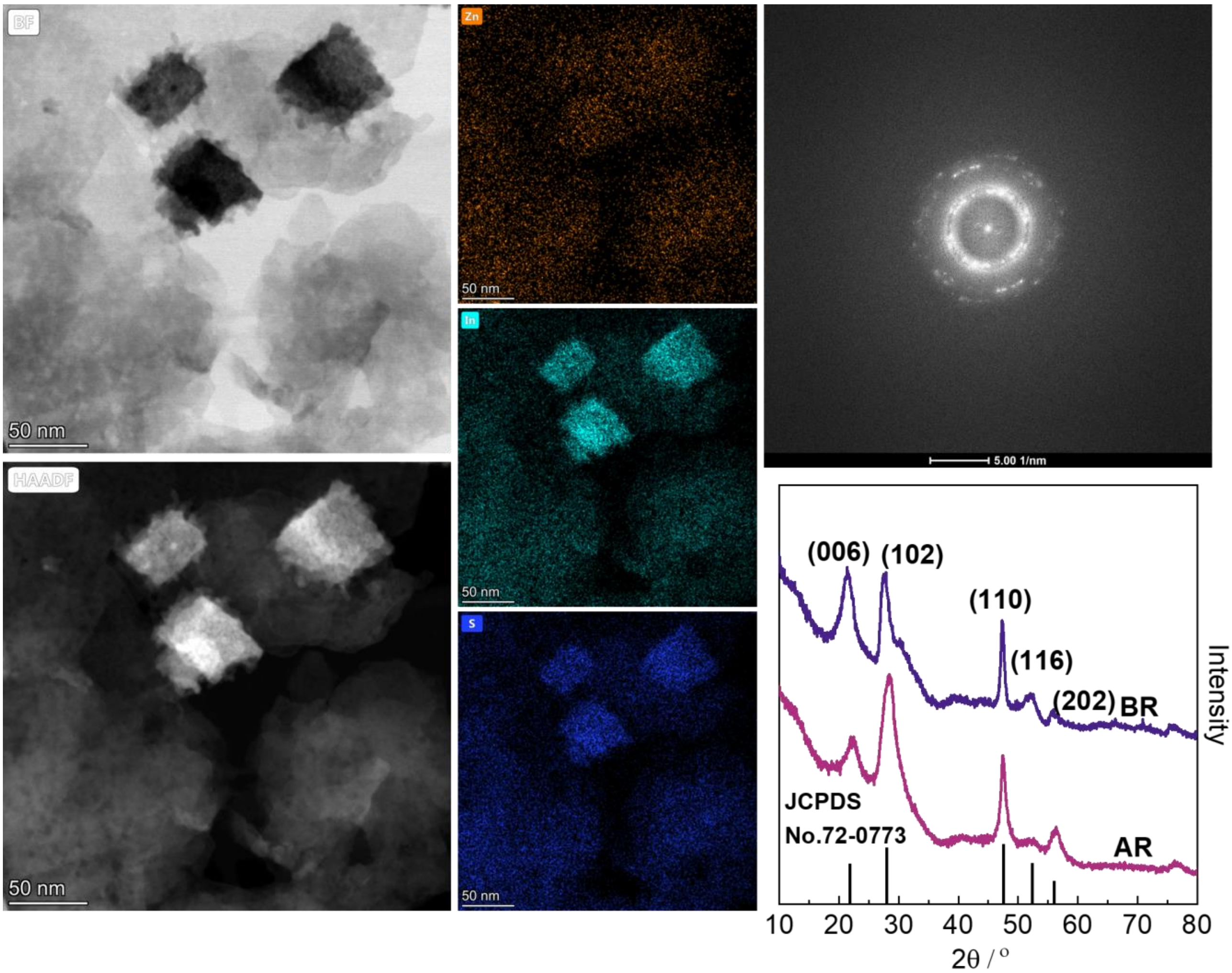

Figure S3. Post catalysis characterizations of ZIS. Bright field and high-angle annular dark-field image in STEM and EDX mapping and the corresponding electron diffraction ring. X-ray diffraction (XRD) of the crystalline ZIS. The diffraction pattern exhibits five characteristic peaks located at 2θ = 21.8°, 27.6°, 47.2°, 52.2°, and 55.7°, corresponding to the (006), (102), (110), (116) and (022) crystal planes of hexagonal ZIS (JCPDS No. 72-0773).[8]

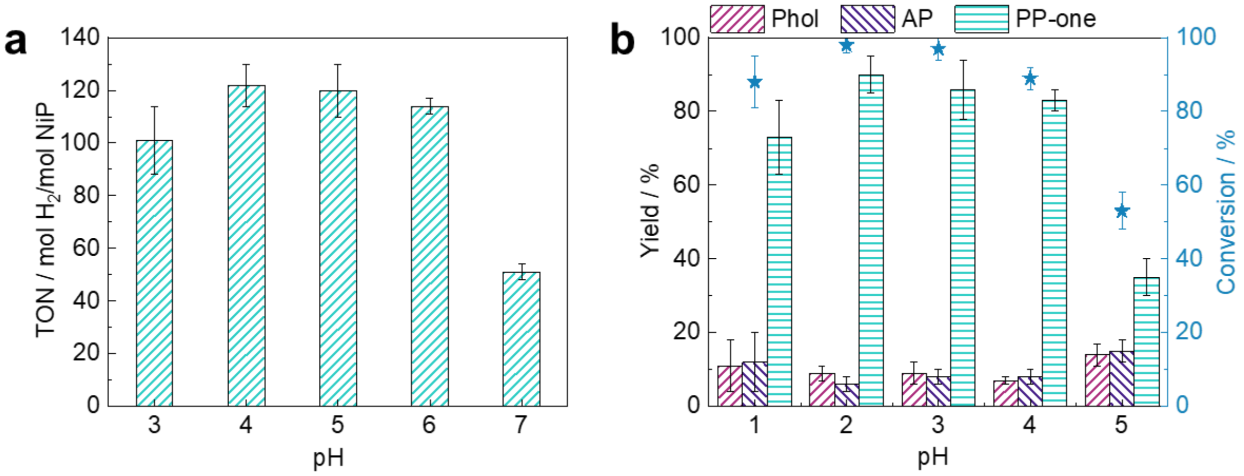

Figure S4. pH effect on H_2 evolution(**a**) and PP-ol conversion (**b**). Reaction condition: PP-ol 3 mg, ZIS (1 mg mL^{-1}), NiP 100 nmol, 1 mL HCl aqueous solution (with different pH), 600 rpm stirring and concentrated AM1.5G irradiation (24 h, 5 sun, 500 mW cm^{-2}).

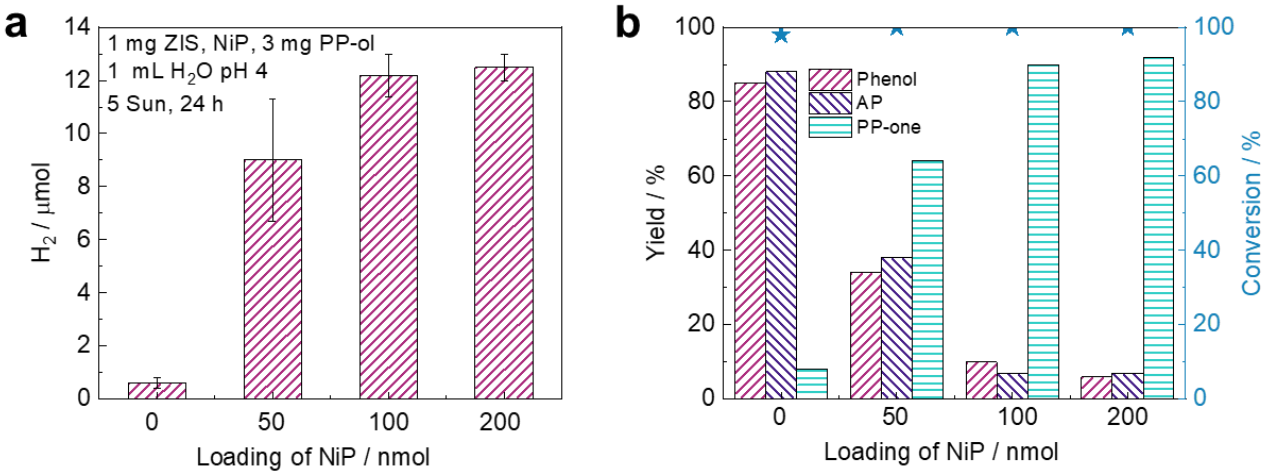

Figure S5. (**a**) Effect of the loading of NiP on H_2 evolution; (**b**) effect of the loading of NiP on PP-ol conversion. Reaction condition: PP-ol (3 mg), ZIS (1 mg mL^{-1}), NiP (loading is 0, 50, 100 or 200 nmol), 1 mL HCl aqueous solution (pH=4), 600 rpm stirring and irradiation (24 h, AM 1.5 G, 500 mW cm^{-2}) without cooling (60±5 °C).

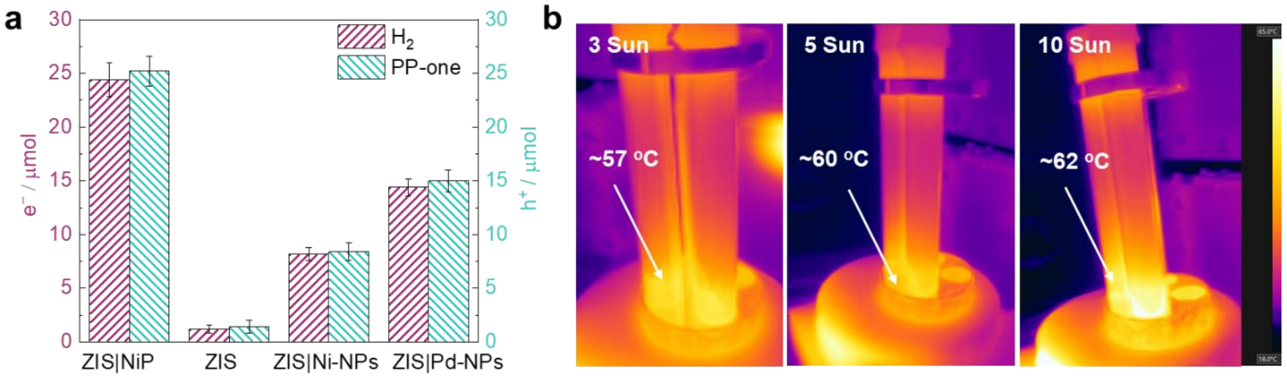

Figure S6. (**a**) Yield of electrons and holes based on H_2 and PP-one production with ZIS|NiP. (**b**) infrared thermal imaging for temperature measurement. Reaction condition: PP-ol 3 mg, ZIS (1 mg mL^{-1}), NiP or other co-catalysts 100 nmol, 1 mL HCl aqueous solution (pH=4), 600 rpm stirring and concentrated AM1.5G irradiation (24 h, 5 sun, 500 mW cm^{-2}).

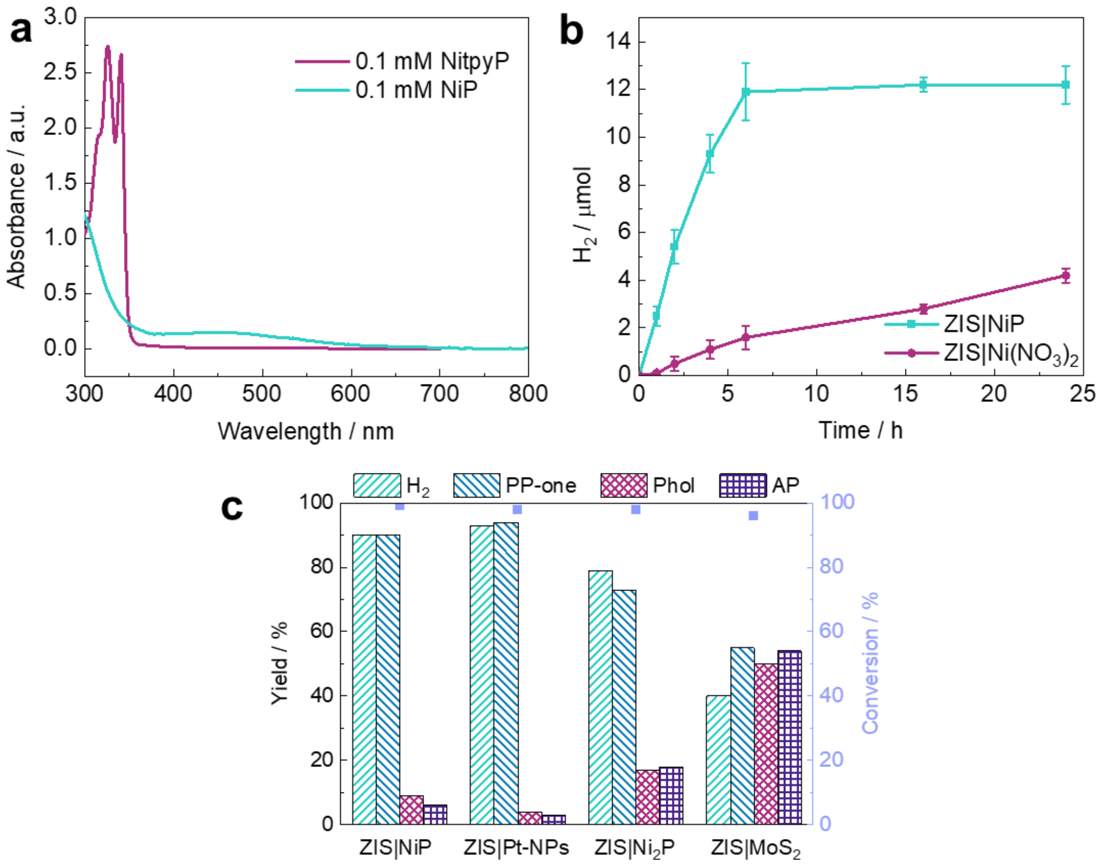

Figure S7. (**a**) UV–vis spectrum of NiP and NitpyP; (**b**) Time profile of photocatalytic H_2 production with ZIS|NiP or ZIS|Ni$(NO_3)_2$. (**c**) Catalytic performance of ZIS|NiP and other catalysts on PP-ol conversion. Reaction condition: PP-ol 3 mg, ZIS (1 mg mL^{-1}), co-catalysts 100 nmol, 1 mL HCl aqueous solution (pH=4), 600 rpm stirring and concentrated AM1.5G irradiation (24 h, 5 sun, 500 mW cm^{-2}).

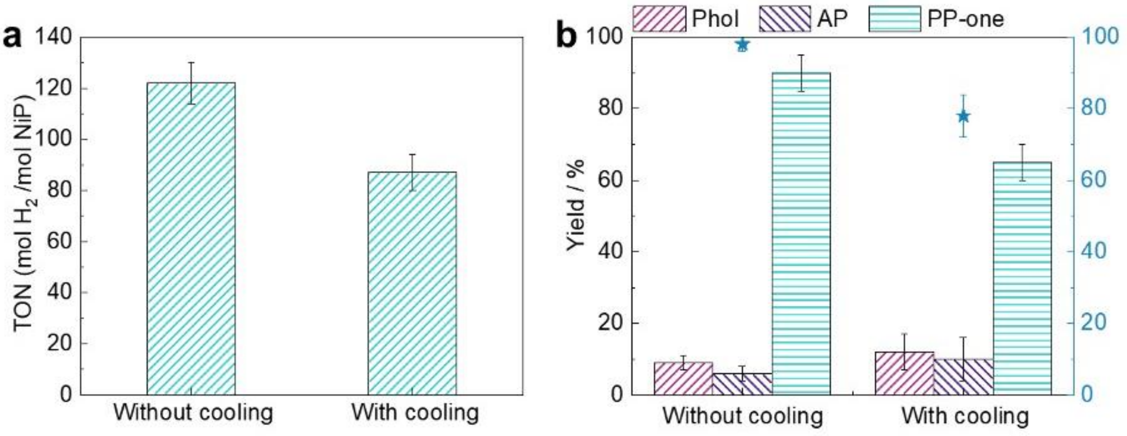

Figure S8. Temperature effect on H_2 evolution (**a**) and PP-ol conversion (**b**). Reaction condition: PP-ol 3 mg, ZIS (1 mg mL^{-1}), NiP 100 nmol, 1 mL HCl aqueous solution (pH=4), 600 rpm stirring and concentrated AM1.5G irradiation (24 h, 5 sun, 500 mW cm^{-2}). Temperature with cooling (25 °C), Temperature without cooling (60±5 °C).

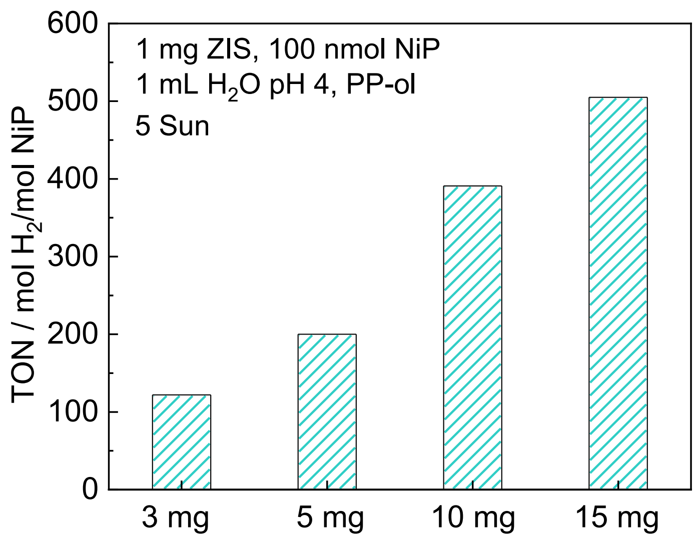

Figure S9. Performance with increasing amounts of PP-ol. Reaction condition: PP-ol (3 mg, 5 mg, 10 mg or 15 mg), ZIS (1 mg mL^{-1}), NiP 100 nmol, 1 mL HCl aqueous solution (pH=4), 600 rpm stirring and concentrated AM1.5G irradiation (24 h, 5 sun, 500 mW cm^{-2}) without cooling (60±5 °C).

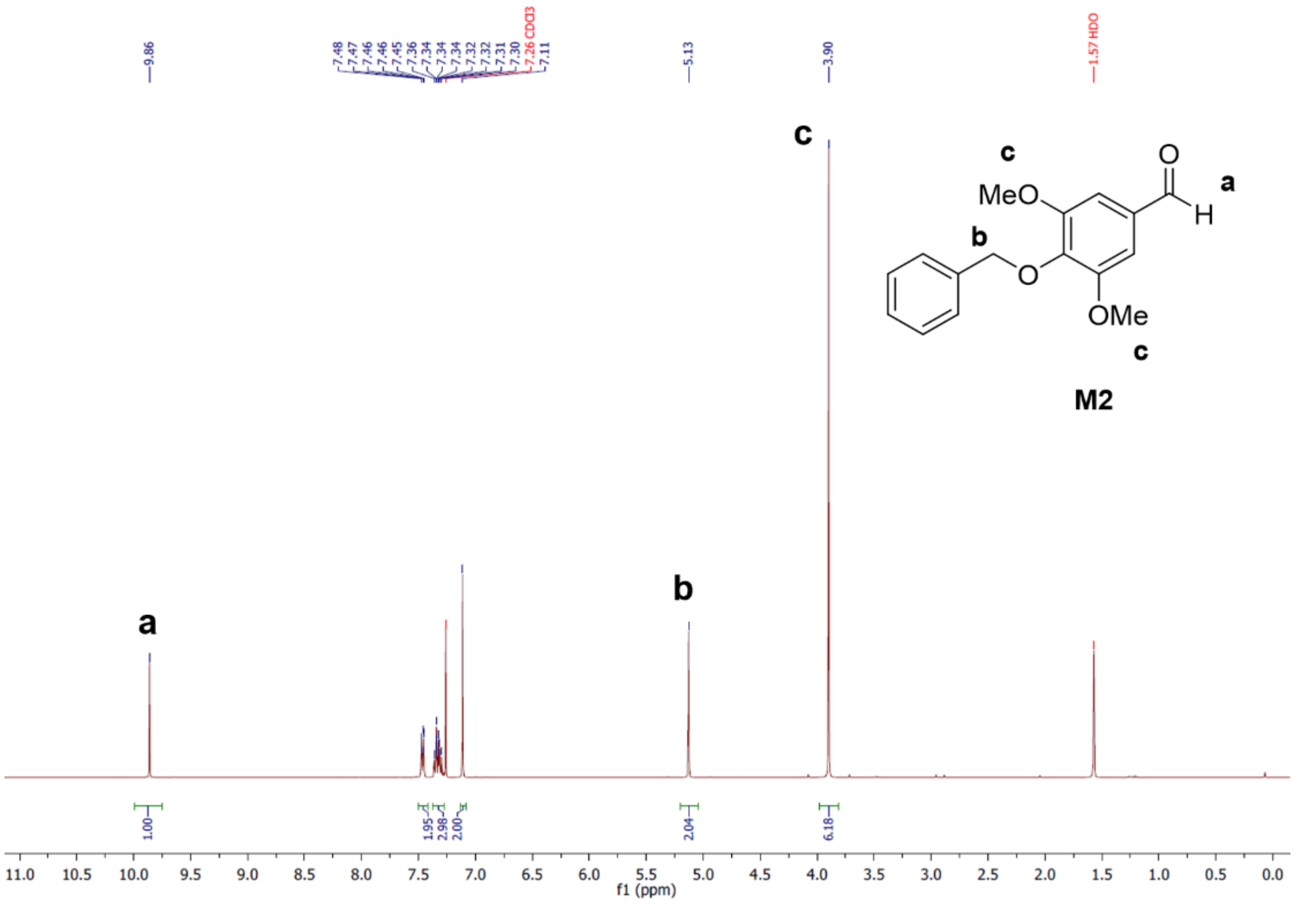

Figure S10. ^{1}H NMR spectrum (400 MHz, $CDCl_3$) of lignin model compound **M2**: δ 9.86 (s, 1H), 7.50 – 7.42 (m, 2H), 7.39 – 7.28 (m, 2H), 7.11 (s, 2H), 5.13 (s, 2H), 3.90 (s, 6H).

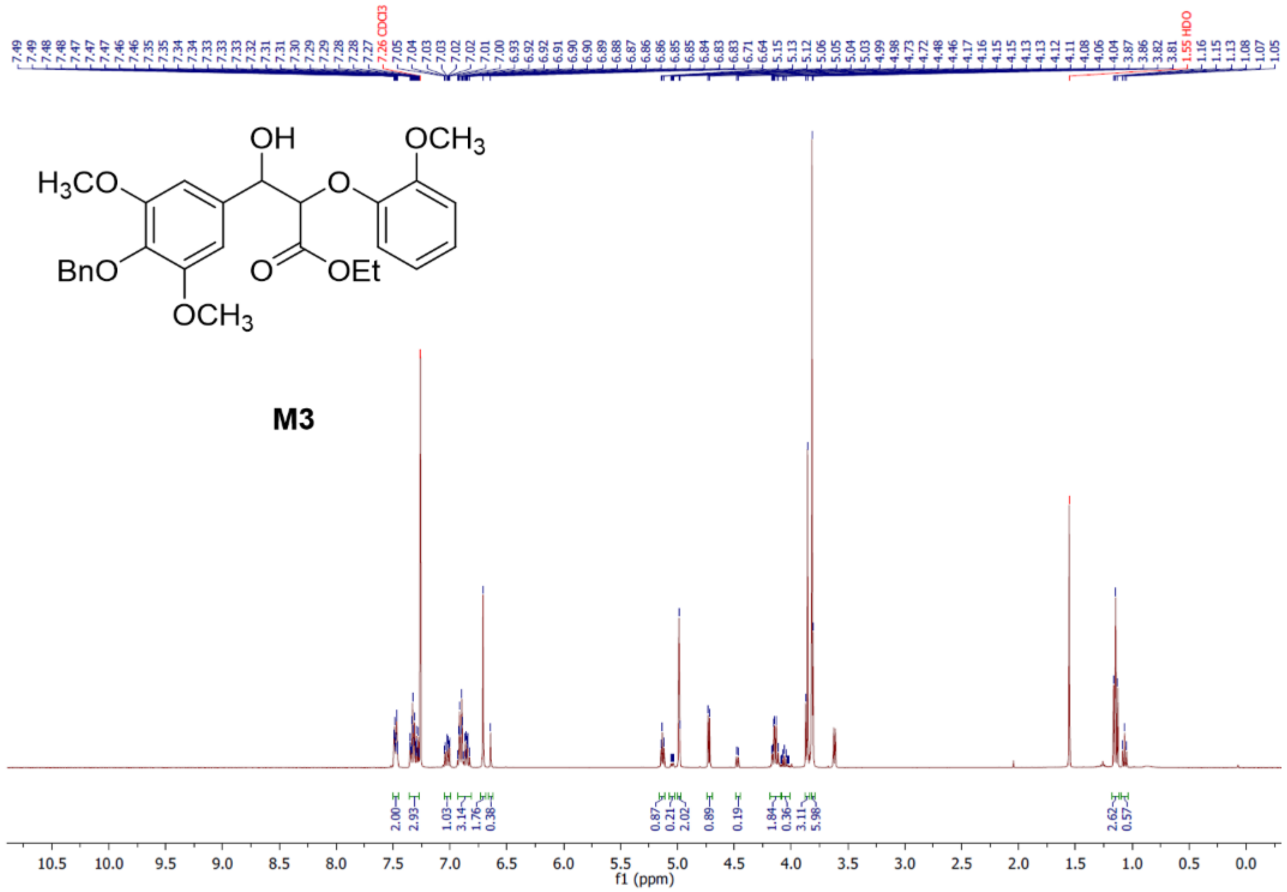

Figure S11. ^{1}H NMR spectrum (400 MHz, $CDCl_3$) of lignin model compound **M3**: δ 7.51 – 7.44 (m, 2H, Ar), 7.40 – 7.27 (m, 3H, Ar), 7.07 – 6.99 (m, 1H, Ar), 6.95 – 6.80 (m, 3H, Ar), 6.71 (s, 2H, Ar, major), 6.64 (s, 2H, Ar, minor), 5.13 (appt, J = 5.4 Hz, 1H, Ar-C*H*-OH, major), 5.04 (appdd, J = 7.0, 3.1 Hz, 1H, Ar-C*H*-OH, minor), 4.99 (s, 2H, -CH_2OPh, major), 4.98 (s, 2H, -CH_2OPh, minor), 4.72 (d, J = 5.2 Hz, 1H, -C*H*OAr, major), 4.47 (d, J = 7.0 Hz, 1H, -C*H*OAr, minor), 4.14 (m, 2H, -OCH_2CH_3, major), 4.09 – 4.02 (m, 2H, -OCH_2CH_3, minor), 3.87 (s, 3H, -OCH_3, minor), 3.86 (s, 3H, -OCH_3, major), 3.82 (s, 6H, , -ArOCH_3, major), 3.81 (s, 6H, ArOCH_3, minor), 1.15 (t, J = 7.1 Hz, 3H, -OCH_2CH_3, major), 1.07 (t, J = 7.1 Hz, 3H, -OCH_2CH_3, minor).

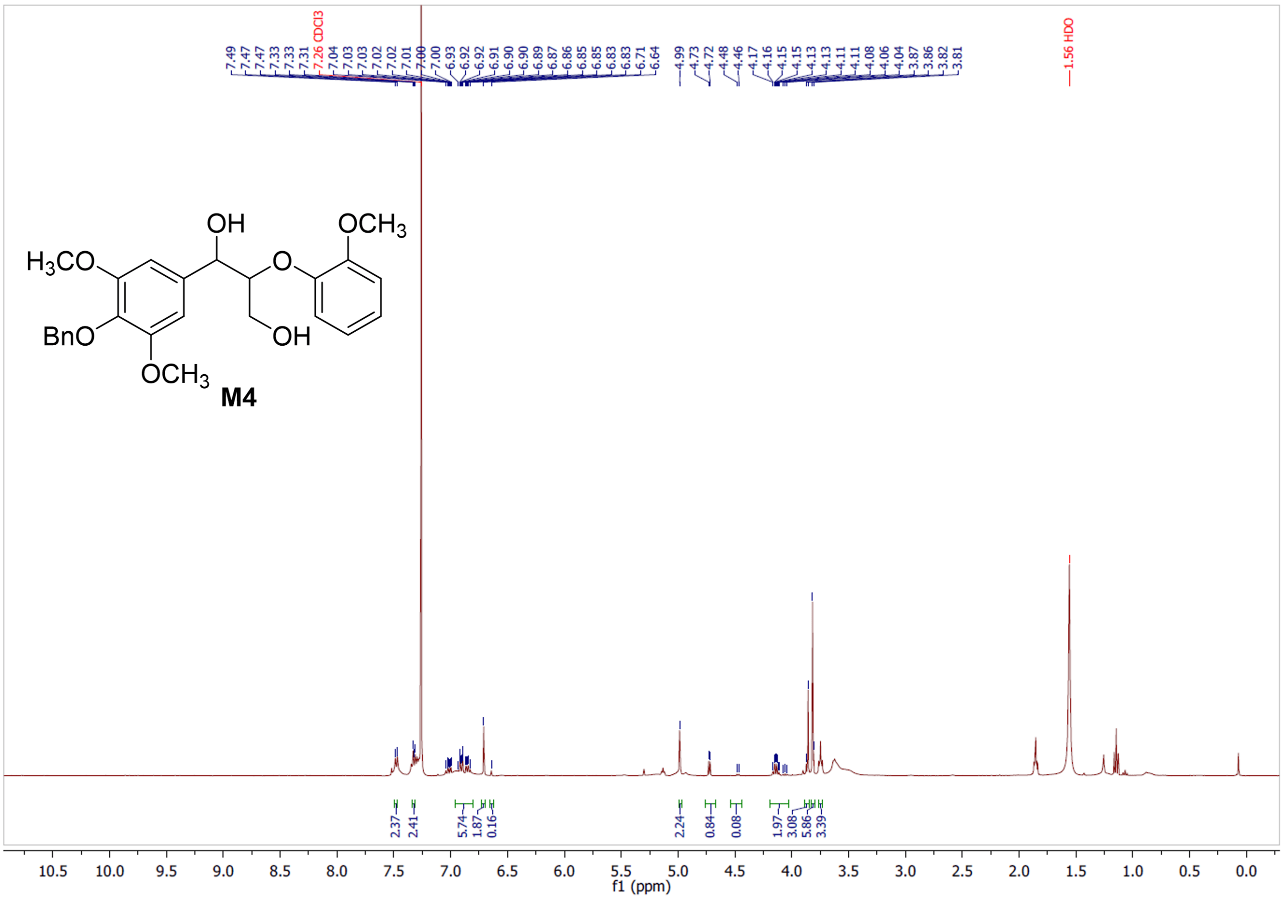

Figure S12. ^{1}H NMR (400 MHz, $CDCl_3$) of lignin model compound **M4**: δ 7.59 – 7.45 (m, Ar, 2H), 7.37 – 7.29 (m, Ar, 2H), 7.12 – 6.77 (m, Ar, 5H), 6.71 (s, 2H, Ar, major), 6.64 (s, 2H, Ar, minor), 4.99 (s, 2H, -C*H*$_2$Ph), 4.72 (d, *J* = 5.2 Hz, 1H, Ar*H*OH, major), 4.47 (d, *J* = 7.0 Hz, 1H, Ar*H*OH, minor), 4.39 – 3.39 (m, 3H, C*H*OAr and C*H*$_2$OH), 3.87 (s, 3H, ArOC*H*$_3$, minor), 3.86 (s, 3H, ArOC*H*$_3$, major), 3.82 (s, 6H, ArOC*H*$_3$, major), 3.81 (s, 3H, minor, ArOC*H*$_3$).

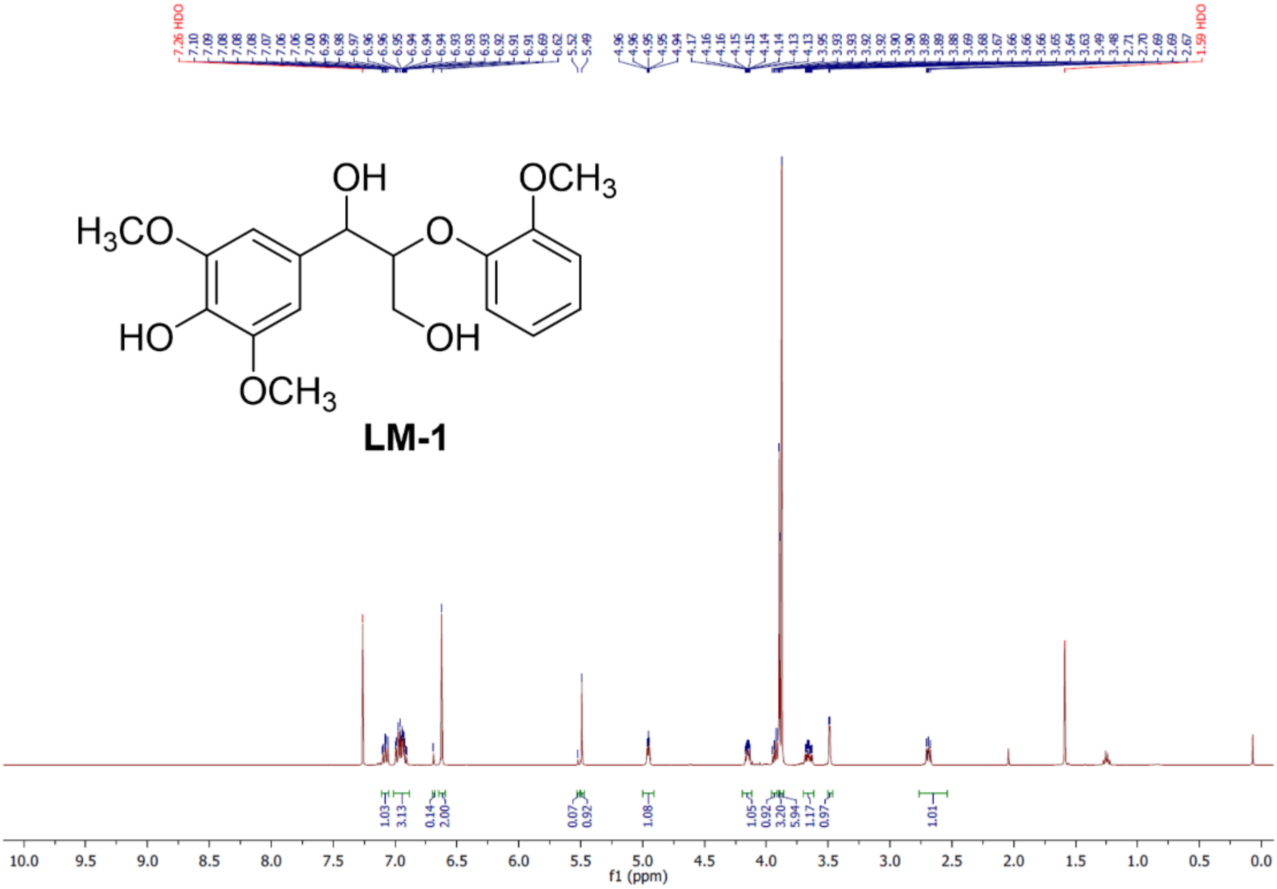

Figure S13. ^{1}H NMR spectrum (400 MHz, $CDCl_3$) of lignin model compound **M5** (**LM-1**): δ 7.18 – 7.02 (m, 1H, Ar), 7.02 – 6.87 (m, 3H, Ar), 6.69 (s, 2H, minor, Ar), 6.62 (s, 2H, major, Ar), 5.52 (s, 1H, -ArO*H*, minor), 5.49 (s, 1H, -ArO*H*, major), 5.01 – 4.91 (m, -C*H*OH, 1H), 4.20 – 4.11 (m, -C*H*OAr, 1H), 3.97 – 3.91 (m, -CH_2OH, 1H), 3.89 (s, ArOCH_3, 3H), 3.88 (s, ArOCH_3, 6H), 3.66 (ddd, J = 12.2, 7.8, 3.4 Hz, -CH_2OH, 1H), 3.49 (d, J = 3.2 Hz, -CHO*H*, 1H), 2.75 – 2.63 (m, -CH_2O*H*, 1H).

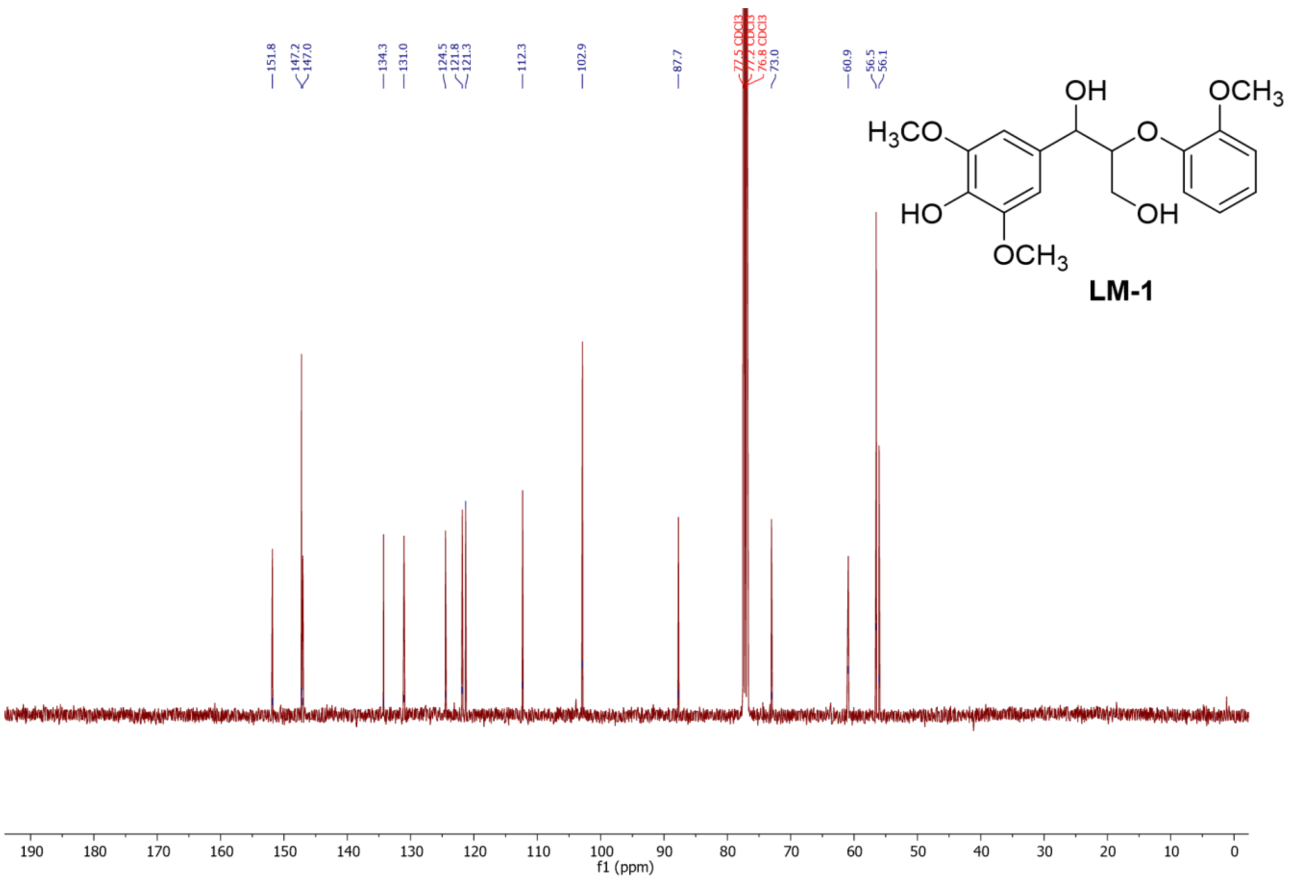

Figure S14. ^{13}C NMR spectrum (101 MHz, $CDCl_3$) of lignin model compounds **M5** (**LM-1**): δ 151.8, 147.2, 147.0, 134.3, 131.0, 124.5, 121.8, 121.3, 112.3, 102.9, 87.7, 73.0, 60.9, 56.5, 56.1.

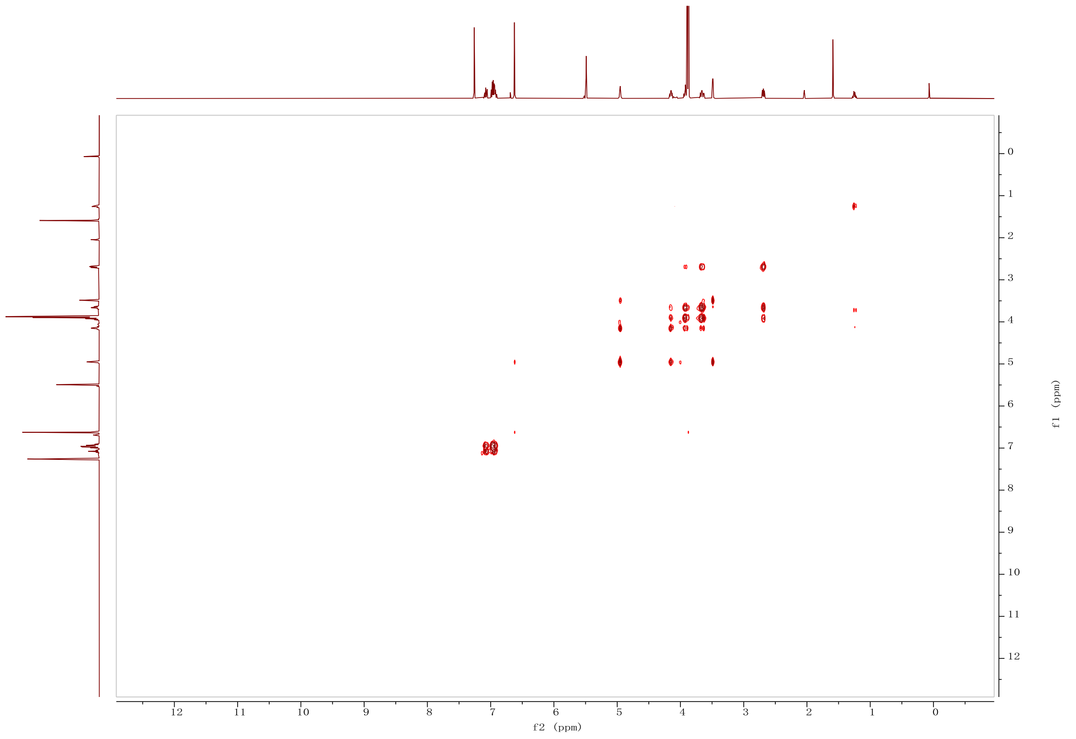

Figure S15. 2D COSY spectrum of lignin model compound **M5** (**LM-1**).

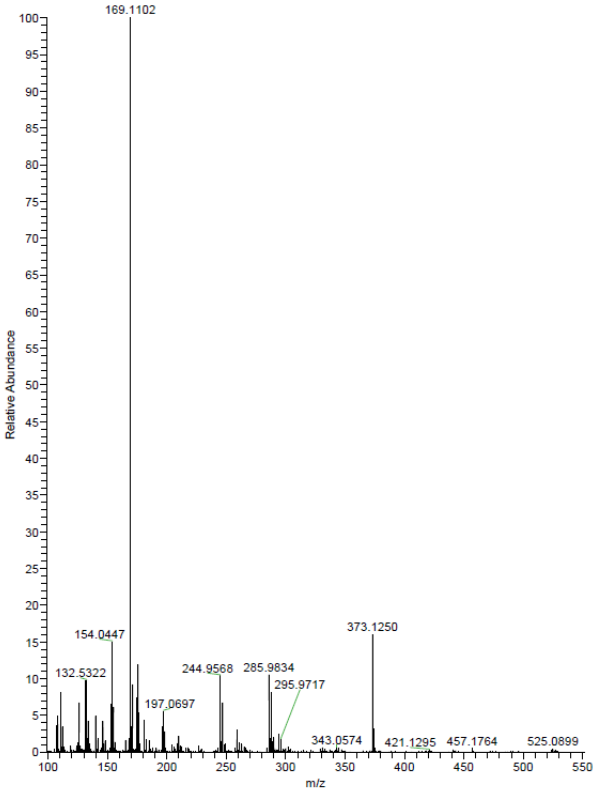

Figure S16. HR-MS spectrum of lignin model compound **M5** (**LM-1**). m/z calcd for $C_{18}H_{22}O_7$ Na: 373.1258, found: 373.1250.

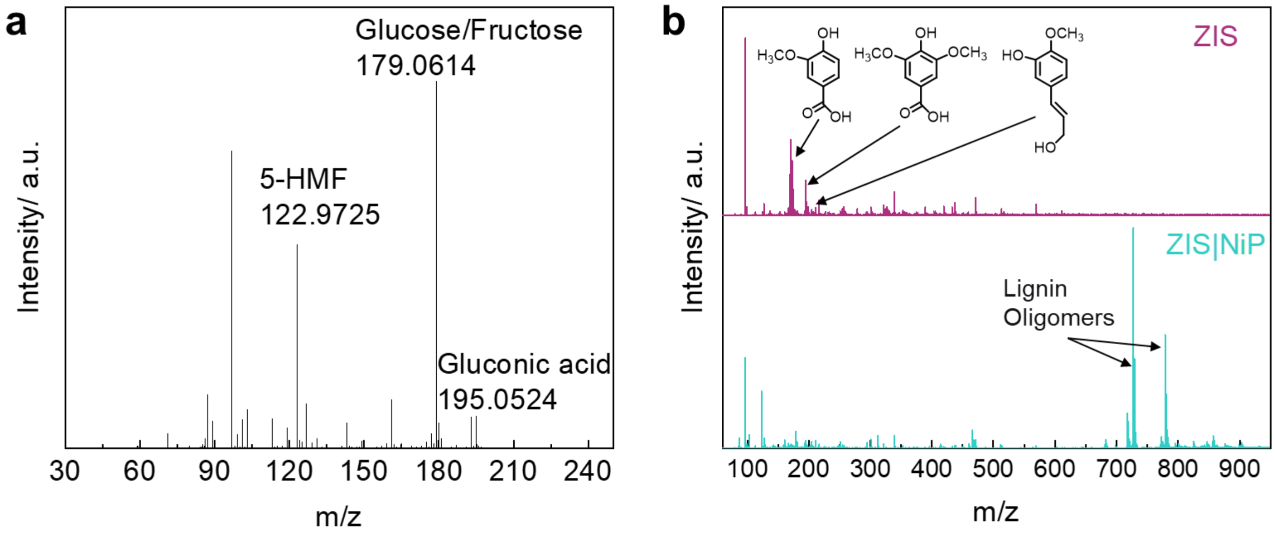

Figure S17. (**a**) LC-MS analysis showing the oxidation products of glucose reforming; (**b**) The product distribution after the sawdust photo-reforming reaction. Reaction condition: glucose/sawdust 3 mg, ZIS (1 mg mL^{-1}), NiP 100 nmol, 1 mL aqueous HCl solution (pH=4), 600 rpm stirring and concentrated AM1.5G irradiation (5 sun, 500 mW cm^{-2}).

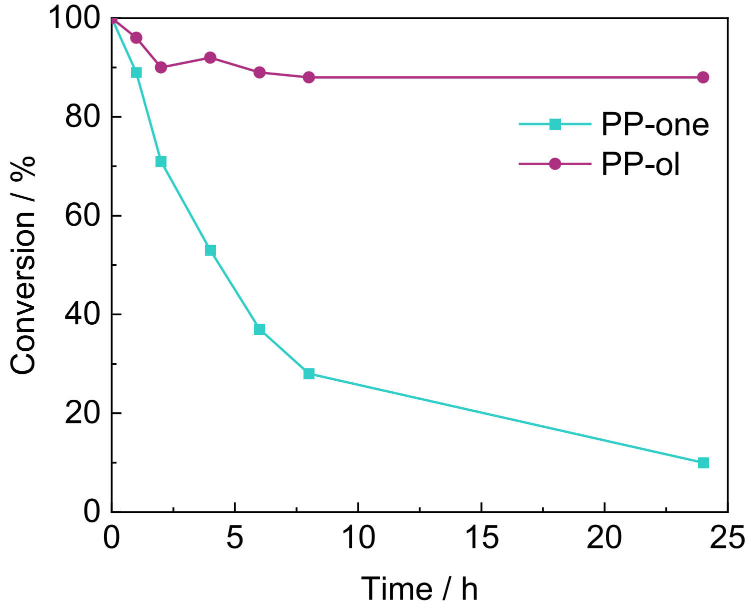

Figure S18. Time profile of conversion of PP-one and PP-ol. Reaction condition: PP-ol 3 mg, TiO_2 (2 mg mL^{-1}), 0.01 mmol of NaOAc, 1 mL EtOH:H_2O (1:9), 600 rpm stirring and concentrated AM1.5G irradiation (24 h, 5 sun, 500 mW cm^{-2}). The proposed mechanism involves photogenerated charge carriers in ZIS, where direct hole transfer from ZIS to PP-ol oxidizes it to PP-one, while photogenerated electrons are transferred from ZIS to NiP, reducing protons to H_2. Cleavage of the β-O-4 linkage requires a bond dissociation energy (BDE) of 54–72 kcal/mol.[9] The oxidation of α carbon atoms in the β-O-4 linkage can efficiently weaken the C-O bond energy by around 14 kcal/mol, making the cleavage process easier[10,11] Consequently, PP-one exhibits a lower BDE than PP-ol, facilitating further C–O bond cleavage. The lower BDE of PP-one was confirmed by using a commercial TiO_2 photocatalyst (Figure S6), where PP-one showed a higher conversion rate than PP-ol. These findings suggest that after solar-driven H_2 production (with H_2 separated in the gas phase), the remaining oxidation products can be further processed to produce aromatic compounds via easier C–O bond cleavage.

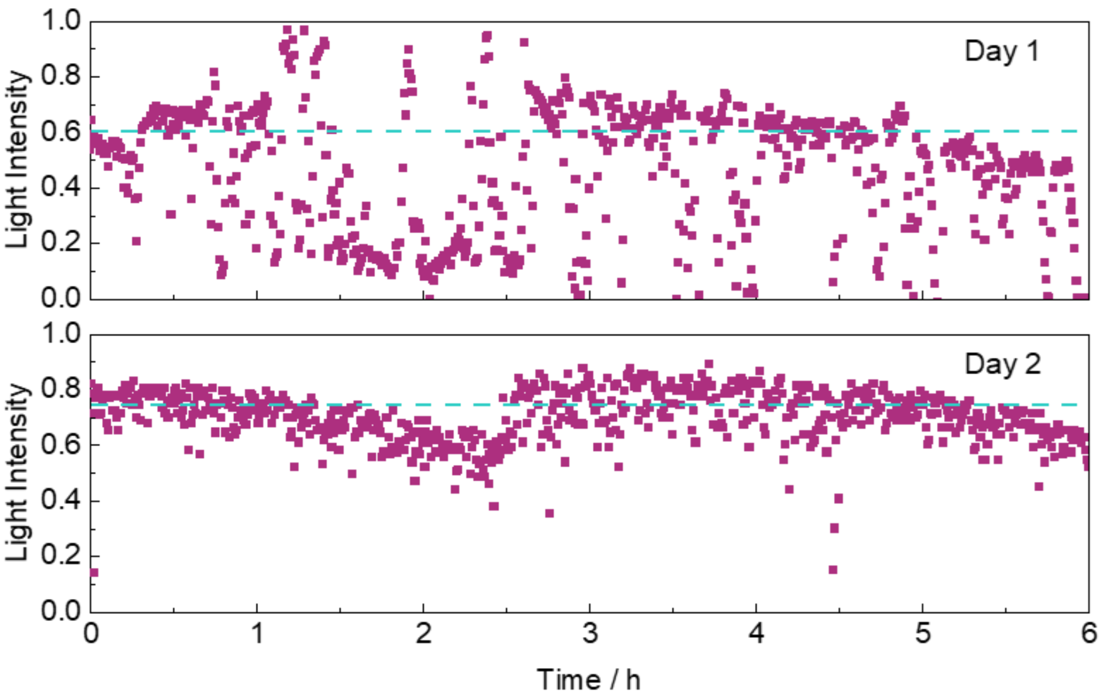

Figure S19. Light intensity during outdoor experiments on the rooftop of the Yusuf Hamied Department of Chemistry, University of Cambridge, on 17th September (day 1) and 11th October 2024 (day 2). Day 1 was a cloudy but warm autumn day, whereas Day 2 was sunny and windy, with typical autumn temperatures. Environmental conditions, including ambient temperature, reaction temperature, and light intensity, were recorded over a 6-hour period from 10:00 AM and 16:00 PM. Light intensity fluctuated between 0-1 Sun due to cloud cover, averaging light intensity of 0.6 Sun on day 1. Light intensity was more stable on Day 2, averaging 0.8 sun throughout the experiment.

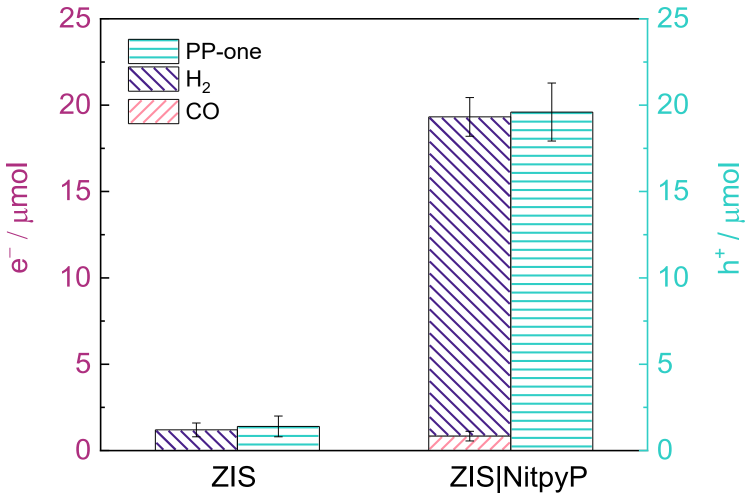

Figure S20. Yield of electrons and holes based on H_2 and PP-one production with ZIS|NiP. Reaction condition: PP-ol 3 mg, ZIS (1 mg mL^{-1}), NitpyP 200 nmol, CO_2-saturated 0.1 M $NaHCO_3$ solution (pH 6.7), 600 rpm stirring and concentrated AM1.5G irradiation (24 h, 5 sun, 500 mW cm^{-2}).

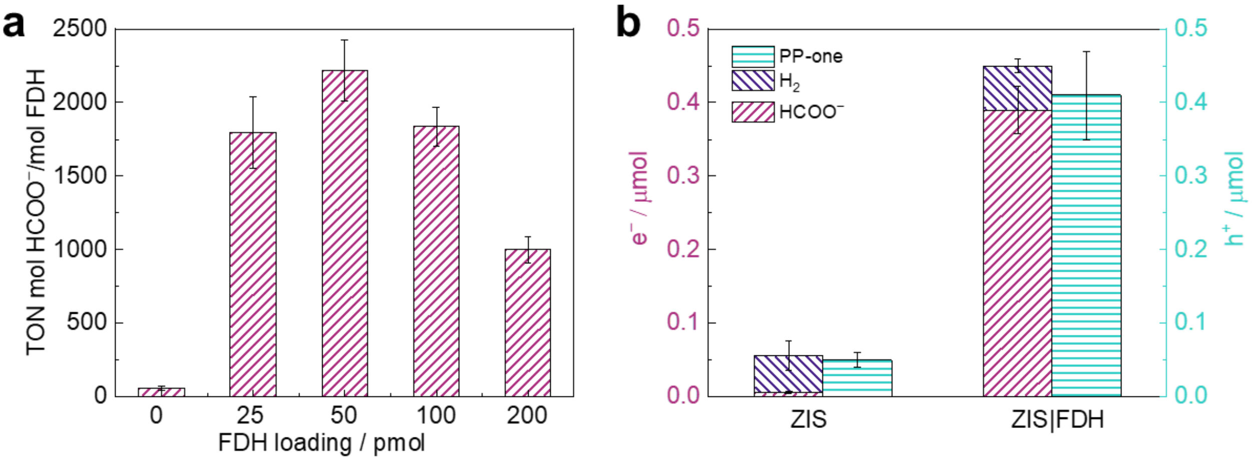

Figure S21. (**a**) Optimization FDH loading for ZIS|FDH catalytic system, reaction condition: PP-ol 1 mg, ZIS (0.5 mg mL^{-1}), FDH (0, 25, 50, 100 or 200 pmol), 1 mL 0.1 M $NaHCO_3$, 600 rpm stirring and irradiation (AM 1.5 G, 100 mW cm^{-2}).; (**b**) Yield of electrons and holes. Reaction condition: PP-ol 1 mg, ZIS (0.5 mg mL^{-1}), FDH 50 pmol, 1 mL 0.1 M $NaHCO_3$, 600 rpm stirring and irradiation (AM 1.5 G, 100 mW cm^{-2}).

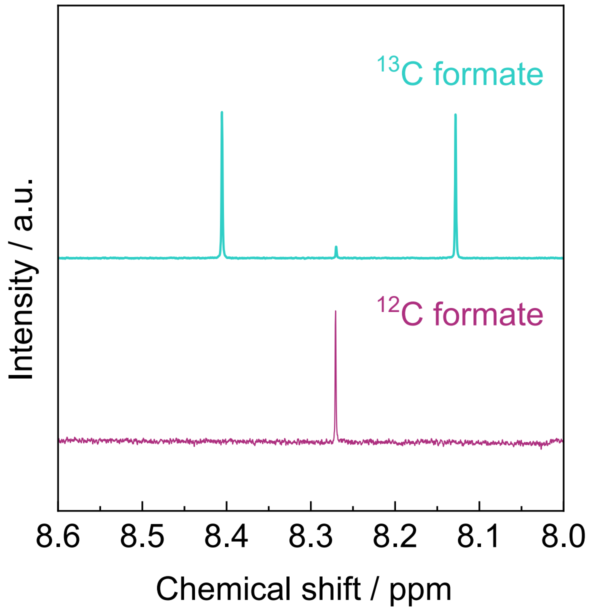

Figure S22. ^{1}H NMR spectra (700 MHz) of the photocatalysis solution after reaction with ZIS|FDH. Isotopic labeling experiments were carried out either in a $NaH^{12}CO_3$ (0.1 M) aqueous solution containing PP-ol (3 mg ml–1) with $^{12}CO_2$ as the headspace gas or in a $NaH^{13}CO_3$ (0.1 M) aqueous solution containing PP-ol (3 mg ml^{-1}) with $^{13}CO_2$ as the headspace gas, under simulated AM 1.5G irradiation. ^{1}H NMR spectra (DMSO-d_6) were collected with a 700 MHz TXO Cryoprobe NMR spectrometer. The ^{1}H NMR spectrum of the photocatalysis solution revealed a doublet $H^{13}COO^{-}$ signal with a coupling constant of 194 Hz in the ^{13}C-labelled sample, and a singlet $H^{12}COO^{-}$ signal at 8.27 ppm in the ^{12}C-labelled sample.

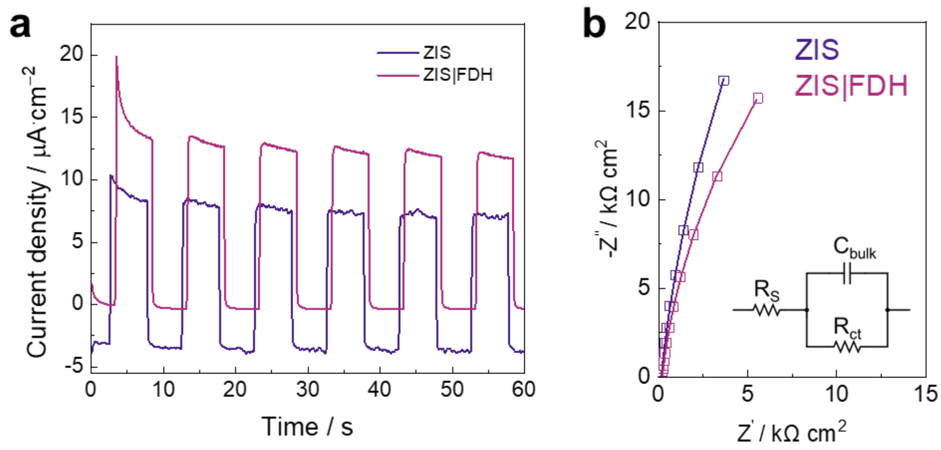

Figure S23. (**a**) Chronoamperometry (CA) of ZIS and ZIS|FDH. Conditions: 20 mL CO_2-saturated 0.1 M $NaHCO_3$ solution (pH 6.7) containing 20 mg PP-ol, Ag/AgCl (sat. KCl) reference electrode, Pt mesh counter electrode, AM 1.5G irradiation, 25 ºC; (**b**) Nyquist plots of photoelectrochemical impedance spectroscopy (PEIS) response (open circuits) with corresponding fitted curves (solid lines). Inset: proposed equivalent circuit to fit the impedance response. Conditions: 20 mL CO_2-saturated 0.1 M $NaHCO_3$ solution (pH 6.7) containing 20 mg PP-ol, Ag/AgCl (sat. KCl) reference electrode, Pt mesh counter electrode, AM 1.5G irradiation, 25 ºC. The ZIS photoelectrode exhibited a photocurrent density of approximately 10 µA cm^{-2} (Supplementary Figure S22), which is suitable for conducting PEIS measurements. The Nyquist plot of the impedance response displays a single semicircle that was fitted using the Randles equivalent circuit comprising a series resistor R_S, a bulk capacitor C_{bulk}, and a charge transfer resistor R_{ct}.

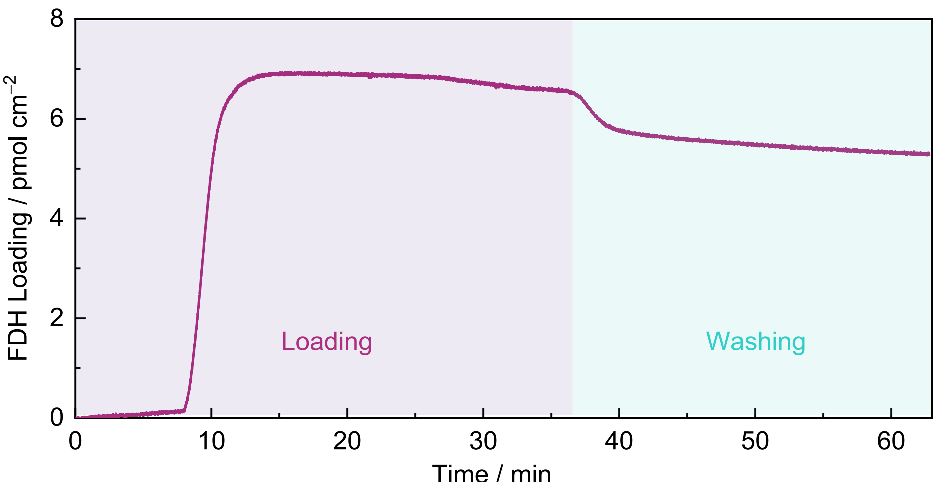

Figure S24. Quartz crystal microbalance (QCM) analysis of the adsorption process of FDH on a ZIS-coated quartz chip. Loading conditions: 0.141 mL min^{-1} flow rate, 2 mL anaerobic $NaHCO_3$ solution (0.1 M, pH 6.7) containing 50 pmol FDH, 25 ºC. Washing conditions: 0.141 mL min^{-1} flow rate, 10 mL anaerobic $NaHCO_3$ solution (0.1 M, pH 6.7), 25 ºC. The interaction between FDH and ZIS was investigated using QCM to evaluate the adsorption and desorption processes of FDH on ZIS. A thin layer of ZIS was deposited onto a gold-coated quartz chip by drop-casting 0.1 ml of an ultrasonicated ZIS suspension (0.5 mg ml^{-1}) in isopropanol. Upon introduction of FDH, the QCM profile exhibits a rapid adsorption phase lasting approximately 6 min, reaching a maximum FDH loading of 6.8 pmol cm^{-2}. Following FDH loading, a washing step was performed at 36 min to assess binding strength. Within 5 min of initiating the washing step, approximately 18% of FDH desorbed, resulting in a final FDH loading of 5.6 pmol cm^{-2}. These QCM results indicate that the association between FDH and ZIS is both rapid and strong.

Supplementary Tables

Table S1. Comparison among Lignin Photoreforming Systems

Catalyst	Substrate	Solvent	Products	Selectivity (%)	TON mol/mol	Product tunability	Ref.
ZIS	PP-ol	H_2O (HCl, pH=4)	AP+Phol	89/88	-	Yes	This work
ZIS|NiP	PP-ol	H_2O (HCl, pH=4)	H_2+PP-one	86/82	120	Yes	This work
CdS	PP-ol	CH_3CN	AP+Phol	>90	3.5 $mmol\cdot g^{-1}\cdot h^{-1}$	-	[12]
CdS/CdO_x	Lignin	10 M KOH	H_2	-	1.3 $mmol\cdot g^{-1}\cdot h^{-1}$	-	[13]
p-CN/P-PDI-3	PP-ol	CH_3CN	BA/PF	59/37	-	-	[14]
mpg-C_3N_4	PP-ol	CH_3CN	BA/PF	51/30	-	-	[15]
MCSCN-75	PP-ol	CH_3CN	BA/PF	85/50	16.7 mg/g	-	[16]
ZIS	PP-ol	CH_3CN/H_2O	AP+Phol	86/82	-	-	[17]
Pd/ZIS+TiO_2	PP-ol	Ethanol	AP+Phol	97/84	-	-	[18]
Ni/CdS	PP-ol	CH_3CN/0.1 M KOH	AP+Phol	97/96	-	Yes	[19]
Ni/CdS	PP-ol	CH_3CN	PP-one	100	-	Yes	[19]
$^{NCN}CN_x$+NiP	Lignin	KPi solution	H_2	-	4	-	[20]
MoS_2/V_S-$ZnIn_2S_4$	Bamboo	H_2O	H_2	-	133 $\mu mol\cdot g^{-1}\cdot h^{-1}$	-	[21]

PP-ol: 2-phenoxy-1-phenylethanol; AP: acetophenone; Phol: phenol; PP-one: 2-phenoxy-1-phenylethanone; BA: benzaldehyde; PF: phenyl formate.

Table S2. Data shown in Figure 2a.

Entry	Time (h)	Phol (µmol)	Yield (%)	AP (µmol)	Yield (%)	PP-one (µmol)	Yield (%)	H_2 (µmol)	Yield (%)
1	1	2.0±0.3	14±2	2.1±0.4	15±3	0.1±0.1	1±1	0.1±0.1	1±1
2	2	4.3±0.1	31±1	4.6±0.1	33±1	0.3±0.3	2±2	0.3±0.3	2±2
3	4	8.0±0.4	57±2	8.1±0.6	58±3	0.5±0.1	4±1	0.4±0.1	3±1
4	6	11.2±0.7	80±5	11.5±1.0	82±7	0.7±0.3	5±2	0.7±0.1	5±1
5	16	11.9±0.7	85±5	12.3±0.8	88±6	0.7±0.3	5±2	0.7±0.3	4±2
6	24	12.5±0.7	89±5	12.3±0.6	88±3	0.7±0.3	5±2	0.7±0.1	5±1

Reaction condition: PP-ol 3 mg, ZIS (1 mg mL^{-1}), 1 mL HCl aqueous solution (pH=4), 600 rpm stirring and irradiation (AM 1.5 G, 500 mW cm^{-2}). $\phi_{overall(Phol+AP)}$ is 1.2%.

Table S3. Data shown in Figure 2b.

Entry	Time (h)	Phol (μmol)	Yield (%)	AP (μmol)	Yield (%)	PP-one (μmol)	Yield (%)	H_2 (μmol)	Yield (%)
1	1	0.4±0.1	3±1	0.4±0.1	3±1	2.9±0.7	21±5	2.7±0.6	19±4
2	2	0.7±0.1	5±1	0.4±0.1	3±1	5.6±0.8	40±6	5.5±0.7	39±5
3	4	1.0±0.3	7±2	1.1±0.4	8±3	9.4±1.1	67±8	9.2±0.8	66±6
4	6	1.1±0.4	8±3	1.0±0.6	7±4	11.8±1.4	84±10	11.9±1.4	85±9
5	16	1.1±0.1	8±1	1.1±0.1	8±1	12.2±0.1	87±1	12.2±0.3	87±2
6	24	1.4±0.6	10±4	1.3±0.4	9±3	12.6±0.5	90±4	12.2±0.8	87±6

Reaction condition: PP-ol 3 mg, ZIS (1 mg mL^{-1}), NiP 100 nmol, 1 mL HCl aqueous solution (pH=4), 600 rpm stirring and irradiation (AM 1.5 G, 500 mW cm^{-2}). $\phi_{overall(H2)}$ is 1.3%.

Table S4. Control experiments.

Entry	Catalyst	Light intensity	H_2 (μmol)	TON (mol H_2/mol NiP)	TOF (mol H_2/mol NiP/h)
1	NiP	5 sun	0±0	0±0	0±0
2	ZIS	5 sun	0.6±0.2	6±2	0.3±0.1
3	ZIS|NiP	-	0±0	0±0	0±0
4[a]	ZIS|NiP	-	0±0	0±0	0±0
5	NitpyP	5 sun	0±0	0±0	0±0

Reaction condition: PP-ol 3 mg, ZIS (1 mg mL^{-1}), NiP 100 nmol, 1 mL HCl aqueous solution (pH=4), 600 rpm stirring and irradiation (24 h, AM 1.5 G, 500 mW cm^{-2}). [a] at 70 °C.

Table S5. Data shown in Figure 2c.

Entry	Catalyst	Phol (µmol)	Yield (%)	AP (µmol)	Yield (%)	PP-one (µmol)	Yield (%)	Conversion (%)
1	ZIS|NiP	1.3±0.3	9±2	0.8±0.3	6±2	12.6±0.7	90±5	98±2
2	ZIS	11.9±0.7	85±5	12.3±0.8	88±6	0.7±0.3	5±2	98±2
3	ZIS|Ni-NPs	4.6±0.4	33±3	4.9±0.6	35±4	4.1±0.4	29±3	67±5
4	ZIS|Pd-NPs	6.2±0.8	44±6	6.3±0.6	45±4	7.7±1.0	55±7	96±4

Reaction condition: PP-ol 3 mg, ZIS (1 mg mL^{-1}), NiP 100 nmol, 1 mL HCl aqueous solution (pH=4), 600 rpm stirring and irradiation (24 h, AM 1.5 G, 500 mW cm^{-2}).

Table S6. Data shown in Figure 2d.

Entry	Catalyst	Light intensity	H_2 (µmol)	TON (mol H_2/mol NiP)	TOF (mol H_2/mol NiP/h)
1	ZIS|NiP	0 sun (Dark)	0.1±0.1	-	-
2	ZIS|NiP	0.5 sun	1.8±0.4	18±4	0.8±0.2
3	ZIS|NiP	1 sun	7.5±1.1	75±11	3.1±0.5
4	ZIS|NiP	5 sun	12.2±0.8	122±8	5.1±0.3
5	ZIS|NiP	10 sun	12.5±0.3	125±3	5.2±0.1

Entry	Light intensity	Phol (µmol)	Yield (%)	AP (µmol)	Yield (%)	PP-one (µmol)	Yield (%)	Conversion (%)
1	0 sun (Dark)	0	0	0	0	0	0	0
2	0.5 sun	2.1±0.7	15±5	2.0±0.6	14±4	1.7±0.6	12±4	30±7
3	1 sun	4.9±0.6	35±4	5.0±0.4	36±3	6.6±0.8	47±7	87±7
4	5 sun	1.3±0.3	9±2	0.8±0.3	6±2	12.6±0.7	90±5	98±2
5	10 sun	0.8±0.4	6±3	1.0±0.3	7±2	12.9±0.4	92±3	100±0

Reaction condition: PP-ol 3 mg, ZIS (1 mg mL^{-1}), NiP, 1 mL HCl aqueous solution (pH=4), 600 rpm stirring and irradiation (24 h, AM 1.5 G; 0, 50, 100, 500 or 1000 mW cm^{-2}).

Table S7. Data shown in Figure 2e.

Entry	Substrates	H_2 (μmol)	TON (mol H_2/mol NiP)	TOF (mol H_2/mol NiP/h)
1	Glucose	10.8±0.4	108±4	4.5±0.2
2	Organosolv lignin	1.8±0.2	18±2	0.8±0.1
3	Sawdust	3.0±0.5	30±5	1.3±0.2
4	LM-1	5.5±4.0	55±4	2.3±0.2
5	LM-2	4.0±0.5	40±5	1.7±0.2

LM-1: 1-(4-hydroxy-3,5-dimethoxyphenyl)-2-(2-methoxyphenoxy)-1,3-propanediol

LM-2: 1-(3,4-dimethoxyphenyl)-2-(2-methoxyphenoxy)-1,3-propanediol (CAS: 10535-17-8)

Reaction condition: Substrate 3 mg, ZIS (1 mg mL^{-1}), NiP 100 nmol, 1 mL HCl aqueous solution (pH=4), 600 rpm stirring and concentrated AM1.5G irradiation (24 h, 5 sun, 500 mW cm^{-2}).

Table S8. Data shown in Figure 2f.

Entry	Scavenger	H_2 (μmol)	TON (mol H_2/mol NiP)	TOF (mol H_2/mol NiP/h)
1	-	12.2±0.8	122±8	5.1±0.3
2	$Na_2S_2O_8$	2.2±0.2	22±2	0.9±0.1
3	$Na_2S + Na_2SO_3$	0.1±0.1	1±1	0±0
4	DMPO	2.7±0.4	27±4	1.1±0.2

Entry	Scavenger	Phol (μmol)	Yield (%)	AP (μmol)	Yield (%)	PP-one (μmol)	Yield (%)	Conversion (%)
1	-	1.3±0.3	9±2	0.8±0.3	6±2	12.6±0.7	90±5	98±2
2	$Na_2S_2O_8$	0.7±0.3	5±2	1.0±0.3	7±2	8.4±0.7	60±5	68±5
3	$Na_2S+Na_2SO_3$	0.3±0.1	2±1	0.1±0.1	1±1	0.1±0.1	1±1	3±1
4	DMPO	0.6±0.1	4±1	0.8±0.3	5±2	1.8±0.3	13±2	20±3

Reaction condition: PP-ol 3 mg, ZIS (1 mg mL^{-1}), NiP 100 nmol, 1 mL HCl aqueous solution (pH=4), Scavenger, 600 rpm stirring and concentrated AM1.5G irradiation (24 h, 5 sun, 500 mW cm^{-2}).

Table S9. Data shown in Figure 3c.

Entry	Catalyst	Day	H_2 (μmol)	TON (mol H_2/mol NiP)	TOF (mol H_2/mol NiP/h)
1	ZIS|NiP	Day 1	11.2	112	18.7
2	ZIS|NiP	Day 2	10.0	100	16.7
3	ZIS	Day 1	0.5	5	0.8
4	ZIS	Day 2	0.3	3	0.5

Entry	Catalyst	Day	Phol (μmol)	Yield (%)	AP (μmol)	Yield (%)	PP-one (μmol)	Yield (%)	Conv. (%)
1	ZIS|NiP	Day 1	1.0	7	1.1	8	12.3	88	94
2	ZIS|NiP	Day 2	1.3	9	1.4	10	10.9	78	87
3	ZIS	Day 1	11.2	80	11.6	83	0.6	4	90
4	ZIS	Day 2	10.6	76	10.9	78	0.4	3	85

Reaction condition: PP-ol 3 mg, ZIS (1 mg mL^{-1}), NiP 100 nmol, 1 mL HCl aqueous solution (pH=4), 600 rpm stirring, 6 h.

Table S10. Data shown in in Figure 4a.

T (h)	Phol (μmol)	Yield (%)	AP (μmol)	Yield (%)	PP-one (μmol)	Yield (%)	H_2 (μmol)	Yield (%)	CO (μmol)	Yield (%)
1	1.1±0.4	8±3	1.1±0.3	8±2	1.3±0.7	9±5	1.1±0.5	8±4	0±0	0±0
2	2.0±0.1	14±1	1.8±0.1	13±1	3.5±0.4	25±3	3.2±0.3	23±2	0±0	0±0
4	2.5±0.8	18±6	2.5±0.4	18±3	6.6±1.1	47±8	6.4±0.8	46±6	0.1±0.1	1±1
6	2.8±0.4	20±3	2.7±0.5	19±4	8.4±1.1	60±8	8.3±1.3	59±9	0.3±0.1	2±1
16	3.6±0.3	26±2	3.9±0.1	28±1	9.2±0.1	66±1	9.0±0.3	64±2	0.4±0.1	3±1
24	3.5±0.6	25±4	4.1±0.3	29±2	9.8±0.8	70±6	9.2±0.5	66±4	0.4±0.1	3±1

Reaction condition: PP-ol 3 mg, ZIS (1 mg mL^{-1}), NitpyR 200 nmol, CO_2-saturated 0.1 M $NaHCO_3$ solution (pH 6.7), 600 rpm stirring and concentrated AM1.5G irradiation (24 h, 5 sun, 500 mW cm^{-2}). $\phi_{overall(H2+CO)}$ is 0.9%.

Table S11. Data shown in Figure 4b.

Entry	Catalyst	CO (μmol)	TON (mol CO/mol MtpyR)	H_2 (μmol)	TON (mol H_2/mol MtpyR)
1	ZIS|NitpyP	0.4±0.1	2±0.7	9.2±0.6	46±3
2	ZIS|CotpyP	0.4±0.1	2±0.5	2.8±0.2	14±1
3	ZIS|CotpyNH$_2$	0.1±0.02	0.5±0.1	1.6±0.4	8±2
4	ZIS|CotpyHCOOH	0.2±0.06	1±0.3	2.6±0.6	13±3

Entry	Catalyst	Phol (μmol)	Yield (%)	AP (μmol)	Yield (%)	PP-one (μmol)	Yield (%)	Conversion (%)
1	ZIS|NitpyP	3.2±0.6	23±4	2.8±0.3	20±2	9.8±0.8	70±6	98±2
2	ZIS|CotpyP	6.7±0.7	48±5	6.3±0.7	45±5	3.2±0.6	23±4	70±5
3	ZIS|CotpyNH$_2$	2.7±0.3	19±2	3.2±0.4	23±3	2.1±0.3	15±2	38±3
4	ZIS|CotpyHCOOH	6.0±0.6	43±4	6.2±0.4	44±3	3.2±0.6	23±4	68±2

Reaction condition: PP-ol 3 mg, ZIS (1 mg mL^{-1}), MtpyR 200 nmol, CO_2-saturated 0.1 M $NaHCO_3$ solution (pH 6.7), 600 rpm stirring and concentrated AM1.5G irradiation (24 h, 5 sun, 500 mW cm^{-2}).

Table S12. Data shown in Figure 4c.

Entry	Time (h)	$HCOO^-$ (μmol)	TON (mol $HCOO^-$/mol FDH)	TOF (mol $HCOO^-$/mol FDH/h)
1	1	0.024±0.010	481±204	481±204
2	2	0.050±0.008	1003±155	502±78
3	4	0.111±0.012	2219±233	555±58
4	6	0.144±0.018	2889±366	481±61
5	8	0.156±0.023	3111±451	389±56
6	24	0.185±0.016	3709±317	155±13

Reaction condition: PP-ol 3 mg, ZIS (1 mg mL^{-1}), FDH 50 pmol, CO_2-saturated 0.1 M $NaHCO_3$ solution (pH 6.7), 600 rpm stirring and irradiation (24 h, AM 1.5 G, 100 mW cm^{-2}). $\phi_{overall(HCOO^-)}$ is 0.1%.

Supporting References

(1) Gross, M. A.; Reynal, A.; Durrant, J. R.; Reisner, E. Versatile Photocatalytic Systems for H_2 Generation in Water Based on an Efficient DuBois-Type Nickel Catalyst. *J. Am. Chem. Soc.,* **2014**, *136* (1), 356–366.

(2) Buendia, J.; Mottweiler, J.; Bolm, C. Preparation of Diastereomerically Pure Dilignol Model Compounds. *Chem. Eur. J.,* **2011**, *17* (49), 13877–13882.

(3) Amancha, P. K.; Liu, H. J.; Wei Ly, T.; Shia, K. S. General Approach to 2,3-Dibenzyl-γ-Butyrolactone Lignans: Application to the Total Synthesis of (±)-5′-Methoxyyatein, (±)-5′-Methoxyclusin, and (±)-4′-Hydroxycubebinone. *Eur. J. Org. Chem.,* **2010**, *2010* (18), 3473–3480.

(4) Hanson, S. K.; Wu, R.; Silks, L. A. "Pete." C-C or C-O Bond Cleavage in a Phenolic Lignin Model Compound: Selectivity Depends on Vanadium Catalyst. *Angew. Chem. Int. Ed.,* **2012**, *51* (14), 3410–3413.

(5) Liu, Y.; Rodríguez-Jiménez, S.; Song, H.; Pannwitz, A.; Kim, D.; Coito, A. M.; Manuel, R. R.; Webb, S.; Su, L.; Bonke, S. A.; Milton, R. D.; Pereira, I. A. C.; Bonnet, S.; Hammarström, L.; Reisner, E. Bio-Inspired Self-Assembly of Enzyme-Micelle Systems for Semi-Artificial Photosynthesis. *Angew. Chem. Int. Ed.,* **2025**, *64* (18), e202424222.

(6) Lancefield, C. S.; Ojo, O. S.; Tran, F.; Westwood, N. J. Isolation of Functionalized Phenolic Monomers through Selective Oxidation and C-O Bond Cleavage of the β-O-4 Linkages in Lignin. *Angew. Chem. Int. Ed.,* **2015**, *54* (1), 258–262.

(7) Xu, L.; Deng, X.; Li, Z. Photocatalytic Splitting of Thiols to Produce Disulfides and Hydrogen over PtS/$ZnIn_2S_4$ Nanocomposites under Visible Light. *Appl. Catal. B,* **2018**, *234*, 50–55.

(8) Tian, Q.; Wu, W.; Liu, J.; Wu, Z.; Yao, W.; Ding, J.; Jiang, C. Dimensional Heterostructures of 1D CdS/2D $ZnIn_2S_4$ Composited with 2D Graphene: Designed Synthesis and Superior Photocatalytic Performance. *Dalton Trans.,* **2017**, *46* (9), 2770–2777.

(9) Kim, S.; Chmely, S. C.; Nimlos, M. R.; Bomble, Y. J.; Foust, T. D.; Paton, R. S.; Beckham, G. T. Computational Study of Bond Dissociation Enthalpies for a Large Range of Native and Modified Lignins. *J. Phys. Chem. Lett.,* **2011**, *2* (22), 2846–2852.

(10) Nguyen, J. D.; Matsuura, B. S.; Stephenson, C. R. J. A Photochemical Strategy for Lignin Degradation at Room Temperature. *J. Am. Chem. Soc.*, **2014**, *136* (4), 1218–1221.

(11) Kim, S.; Chmely, S. C.; Nimlos, M. R.; Bomble, Y. J.; Foust, T. D.; Paton, R. S.; Beckham, G. T. Computational Study of Bond Dissociation Enthalpies for a Large Range of Native and Modified Lignins. *J. Phys. Chem. Lett.,* **2011**, *2* (22), 2846–2852.

(12) Wu, X.; Fan, X.; Xie, S.; Lin, J.; Cheng, J.; Zhang, Q.; Chen, L.; Wang, Y. Solar Energy-Driven Lignin-First Approach to Full Utilization of Lignocellulosic Biomass under Mild Conditions. *Nat. Catal.* **2018**, *1* (10), 772–780.

(13) Wakerley, D. W.; Kuehnel, M. F.; Orchard, K. L.; Ly, K. H.; Rosser, T. E.; Reisner, E. Solar-Driven Reforming of Lignocellulose to H_2 with a CdS/CdO_x Photocatalyst. *Nat. Energy,* **2017**, *2* (4), 17021.

(14) Xu, X.; Dai, S.; Xu, S.; Zhu, Q.; Li, Y. Efficient Photocatalytic Cleavage of Lignin Models by a Soluble Perylene Diimide/Carbon Nitride S-Scheme Heterojunction. *Angew. Chem. Int. Ed.,* **2023**, *62* (44), e202309066.

(15) Liu, H.; Li, H.; Lu, J.; Zeng, S.; Wang, M.; Luo, N.; Xu, S.; Wang, F. Photocatalytic Cleavage of C-C Bond in Lignin Models under Visible Light on Mesoporous Graphitic Carbon Nitride through π-π Stacking Interaction. *ACS Catal.,* **2018**, *8* (6), 4761–4771.

(16) Ku, C.; Li, K.; Guo, H.; Wu, Q.; Yan, L. One-Step Construction of Mesoporous Cyano and Sulfur Co-Modified Carbon Nitride for Photocatalytic Valorization of Lignin to Functionalized Aromatics. *Appl. Surf. Sci.,* **2022**, *592*, 153266.

(17) Lin, J.; Wu, X.; Xie, S.; Chen, L.; Zhang, Q.; Deng, W.; Wang, Y. Visible-Light-Driven Cleavage of C−O Linkage for Lignin Valorization to Functionalized Aromatics. *ChemSusChem,* **2019**, *12* (22), 5023–5031.

(18) Luo, N.; Wang, M.; Li, H.; Zhang, J.; Hou, T.; Chen, H.; Zhang, X.; Lu, J.; Wang, F. Visible-Light-Driven Self-Hydrogen Transfer Hydrogenolysis of Lignin Models and Extracts into Phenolic Products. *ACS Catal.,* **2017**, *7* (7), 4571–4580.

(19) Han, G.; Yan, T.; Zhang, W.; Zhang, Y. C.; Lee, D. Y.; Cao, Z.; Sun, Y. Highly Selective Photocatalytic Valorization of Lignin Model Compounds Using Ultrathin Metal/CdS. *ACS Catal.,* **2019**, *9* (12), 11341–11349.

(20) Kasap, H.; Achilleos, D. S.; Huang, A.; Reisner, E. Photoreforming of Lignocellulose into H_2 Using Nanoengineered Carbon Nitride under Benign Conditions. *J. Am. Chem. Soc.,* **2018**, *140* (37), 11604–11607.

(21) Tang, J. P.; Chen, Y.; Wang, Z. Y.; Hu, Y. H.; Wang, J. H.; Bao, L.; Zhao, Z. Y.; Yuan, Y. J. Sustainable H_2 Production from Lignocellulosic Biomass over MoS_2 Modified Sulfur Vacancy Enriched $ZnIn_2S_4$ Photocatalyst. *ACS Catal.,* **2025**, *15* (1), 265–274.

(22) Kuehnel, M. F.; Orchard, K. L.; Dalle, K. E.; Reisner, E. Selective Photocatalytic CO_2 Reduction in Water through Anchoring of a Molecular Ni Catalyst on CdS Nanocrystals. *J. Am. Chem. Soc.,* **2017**, *139* (21), 7217–7223.

(23) Kim, D.; Bhattacharjee, S.; Lam, E.; Casadevall, C.; Rodríguez-Jiménez, S.; Reisner, E. Photocatalytic CO_2 Reduction Using Homogeneous Carbon Dots with a Molecular Cobalt Catalyst. *Small,* **2024**, *20* (39), 2400057.
